# COVID‐19 in early 2023: Structure, replication mechanism, variants of SARS‐CoV‐2, diagnostic tests, and vaccine & drug development studies

**DOI:** 10.1002/mco2.228

**Published:** 2023-04-08

**Authors:** Ilker Polatoğlu, Tulay Oncu‐Oner, Irem Dalman, Senanur Ozdogan

**Affiliations:** ^1^ Department of Bioengineering Manisa Celal Bayar University Yunusemre Manisa Turkey; ^2^ Department of Bioengineering Ege University Bornova Izmir Turkey

**Keywords:** COVID‐19 infection, diagnosis tests, drug, SARS‐CoV‐2, vaccine type, variants

## Abstract

Coronavirus Disease‐19 (COVID‐19) is an infectious disease caused by severe acute respiratory syndrome‐coronaviruses‐2 (SARS‐CoV‐2), a highly pathogenic and transmissible coronavirus. Most cases of COVID‐19 have mild to moderate symptoms, including cough, fever, myalgias, and headache. On the other hand, this coronavirus can lead to severe complications and death in some cases. Therefore, vaccination is the most effective tool to prevent and eradicate COVID‐19 disease. Also, rapid and effective diagnostic tests are critical in identifying cases of COVID‐19. The COVID‐19 pandemic has a dynamic structure on the agenda and contains up‐to‐date developments. This article has comprehensively discussed the most up‐to‐date pandemic situation since it first appeared. For the first time, not only the structure, replication mechanism, and variants of SARS‐CoV‐2 (Alpha, Beta, Gamma, Omicron, Delta, Epsilon, Kappa, Mu, Eta, Zeta, Theta, lota, Lambda) but also all the details of the pandemic, such as how it came out, how it spread, current cases, what precautions should be taken, prevention strategies, the vaccines produced, the tests developed, and the drugs used are reviewed in every aspect. Herein, the comparison of diagnostic tests for SARS‐CoV‐2 in terms of procedure, accuracy, cost, and time has been presented. The mechanism, safety, efficacy, and effectiveness of COVID‐19 vaccines against SARS‐CoV‐2 variants have been evaluated. Drug studies, therapeutic targets, various immunomodulators, and antiviral molecules applied to patients with COVID‐19 have been reviewed.

## Graphical abstract

This paper reviews not only the structure, replication mechanism, and variants of severe acute respiratory syndrome (SARS)‐CoV‐2 but also exposes all the details of the pandemic, such as how it came out, how it spread, what precautions should be taken, and prevention strategies in every aspect. It also compares the molecular and serological assays used for SARS‐CoV‐2 diagnosis and lists the COVID‐19 vaccines approved by the countries.

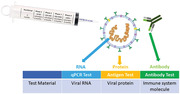

## 1 INTRODUCTION

Respiratory viral diseases such as severe acute respiratory syndrome (SARS) and Middle East respiratory syndrome (MERS) have posed a threat to humans worldwide.^1^ Coronaviruses (CoVs), which cause these diseases, have a size of nearly 65–125 nm in diameter and pertain to the *Coronaviridae* family.^2^ This family, together with the *Roniviridae*, the *Arteriviridae*, and the *Mesoniviridae*, belong to *the Nidovirales* order.^3^ The *Coronaviridae* family includes a single‐stranded ribonucleic acid (ssRNA) that changes size between 20 and 32 kbs in length. Therefore, they are described as large‐genome Nidoviruses.^4^ On the other hand, this family divides into four subgroups that are beta (β), alpha (α), delta (δ), and gamma (γ) CoVs. Only β and α CoVs are recognized to affect humans.^2^ *The Coronaviridae* family is known to be transmitted from animals to humans or zoonotic.^5^ These contain SARS‐CoV and MERS‐CoV that were first recognized in 2003 and 2012, respectively. According to the epidemiological data before 2019, there were six CoVs members infecting humans: MERS‐CoV, SARS‐CoV, NL63 (α coronavirus), OC43 (β coronavirus), HKU1 (β coronavirus), and 229E (α coronavirus).^4^, ^6^, ^7^

For an unknown reason, pneumonia was diagnosed in a few patients in Hubei province, China, in 2019. Several laboratories in China carried out sequencing and etiological investigations for a causative agent of pneumonia. As a result of these investigations, the causative agent of pneumonia was verified as a novel coronavirus. According to the sequence analysis, this new virus pertains to β coronavirus, which contains both SARS‐CoV and MERS‐CoV.^1^

In this review, the status of COVID‐19 in early 2023 has been summarized. First, how COVID‐19 came out, how it spread, precautions to be taken, and prevention strategies have been mentioned, and current cases have been given. The variants of SARS‐CoV‐2 have been given, and it has been mentioned that the Omicron variant and especially its subvariant XBB 1.5 was the most transmissible form. The structure and replication mechanism of SARS‐CoV‐2 have been detailed. The procedure, accuracy rate, cost, sample sources, detection region, and result time of tests used to detect SARS‐CoV‐2 have been compared. The general mechanism of vaccines, including protein‐based, viral‐vector, whole virus, and nucleic acid vaccines, have been explained. The safety, efficacy, and effectiveness of COVID‐19 vaccines against SARS‐CoV‐2 variants have been reviewed, and approved COVID‐19 vaccines have been listed. Various immunomodulators and antiviral molecules applied to patients with COVID‐19 have been evaluated. Viral polymerase, the main protease (M^pro^), and the papain‐like protease (PL^pro^) have been discussed as therapeutic targets. Drug resistance and convalescent plasma and monoclonal antibodies (mAbs) used for COVID‐19 therapies have been detailed.

## 2 CURRENT CASES OF COVID‐19

World Health Organization (WHO) was informed about the cases of pneumonia for unknown reasons in Wuhan City of China on December 31, 2019.^8^ On February 4, 2021, 103,989,900 cases and 2,260,259 deaths were reported worldwide. As of January 21, 2023, 663,640,386 total cases and 6,713,093 total deaths have been reported worldwide. These data showed that the cumulative number of cases had increased more than six times and deaths had increased almost three times in the last 2 years. The current number of cases, deaths, and persons fully vaccinated with the last dose of primary series per 100 population based on continent and country is given in Table 1.^9^

**TABLE 1:** The current numbers based on the continent and country (as of January 21, 2023).

	**Cases** **^a^**	**Deaths** **^a^**	**Vaccinated persons (%)** **^b^**
**Country**			
United States of America	100,304,472	1,088,854	68.42
India	44,681,650	530,728	68.95
France	38,372,611	160,051	78.93
Germany	37,659,518	164,585	76.4
Brazil	36,677,844	695,615	79.52
Japan	31,819,310	64,220	81.55
Republic of Korea	29,955,366	33,134	86.7
Italy	25,363,742	185,993	82.96
The United Kingdom	24,259,240	203,229	74.59
Russian Federation	21,882,414	394,610	54.09
Türkiye	17,004,677	101,419	60.93
Spain	13,711,251	117,759	79.17
Viet Nam	11,526,284	43,186	88.2
Australia	11,269,081	16,989	84,92
China	11,001,439	34,375	86.82
Argentina	10,024,095	130,338	83.72
The Netherlands	8,578,818	22,992	68.69
Iran	7,562,998	144,728	69.68
Mexico	7,314,891	331,595	63.48
Indonesia	6,727,609	160,772	63.14
Poland	6,374,976	118,678	59.72
Colombia	6,354,791	142,385	72.56
Austria	5,749,735	21,595	75.04
Greece	5,683,897	35,392	72.22
Portugal	5,562,214	25,942	86.33
Ukraine	5,367,032	110,978	34.65
Chile	5,096,886	63,599	92.57
Malaysia	5,033,943	36,923	85.07
**Continent**			
Europe	271,197,645	2,174,093	64.49
Americas	187,862,206	2,902,348	70.79
Western Pacific	111,125,686	308,060	84.91
South‐East Asia	60,751,075	803,537	68.11
Eastern Mediterranean	23,236,089	349,221	48.24
Africa	9,466,921	175,177	27.85
**GLOBAL**	**663,640,386**	**6,713,093**	**64.61**

^a^ Cumulative total.

^b^ Persons fully vaccinated with the last dose of primary series per 100 population.

## 3 VARIANTS OF SARS‐CoV‐2

All viruses, including SARS‐CoV‐2, naturally change or mutate over time. Most of these changes have minimal impact on the virus's properties, while some could affect the virus's characteristics. As a result of mutations, change in the spreading rate or the severity of the associated disease could be observed. Also, the efficacy of therapeutic drugs and vaccines could change.^10^ Variants of concern (VOC) and variants of interest (VOI) are two main classifications used to assess the virological and clinical relevance of different variants.^11^ As of May 15, 2022, SARS‐CoV‐2 variants that meet the definition of a VOC are the Alpha variant (Lineage B.1.1.7), Beta variant (Lineage B.1.351), Gamma variant (Lineage P1), Omicron variant (Lineage B.1.1.529), and Delta variant (Lineage B.1.617.2). The variants that meet the definition of a VOI are the Epsilon variant (Lineage B.1.427 and B.1.429), Kappa variant (B.1.617.1), Mu variant (Lineage B.1.621), Eta variant (B.1.525), Zeta variant (Lineage P.2), Theta variant (P.3), lota variant (B.1.526), and Lambda variant (Lineage C.37).^12^ Currently, circulating VOC are only the Omicron variant, while the Beta, Alpha, Gamma, and Delta variants are previously circulating variants. Also, there is no currently circulating variant of interest. The Omicron variant's transmission, reported on November 11, 2021, is hugely higher than previously identified SARS‐CoV‐2 variants.^11^ This is due to the greater ability of this variant to evade the immune system rather than higher viral loads.^13^ As of October 12, 2022, Omicron subvariants under monitoring were BA.5, BA.2.75, BJ.1, BA.4.6, XBB, and BA.2.3.20. On the other hand, as of January 13, 2023, Omicron subvariants under monitoring are BF.7, BQ.1, BA.2.75, and XBB. XBB, which is a recombinant of BJ.1 and BA.2.75, is currently being seen across many countries. Especially its subvariant XBB 1.5 is thought to be the most transmissible variant to date.^12^ This variant is a less severe disease (risk of hospitalization and death), compared with the others owing to less lower respiratory tract replication.^14^ A study reported that the Alpha variant carries a 48% higher risk of serious disease than wild‐type variants. On the other hand, it was stated that the Beta variant has a 24% higher risk of serious disease, compared to the Alpha variant, and 57% of COVID‐19‐related deaths were caused by this variant.^15^

## 4 STRUCTURE AND REPLICATION MECHANISM OF SARS‐CoV‐2

**FIGURE 1:**
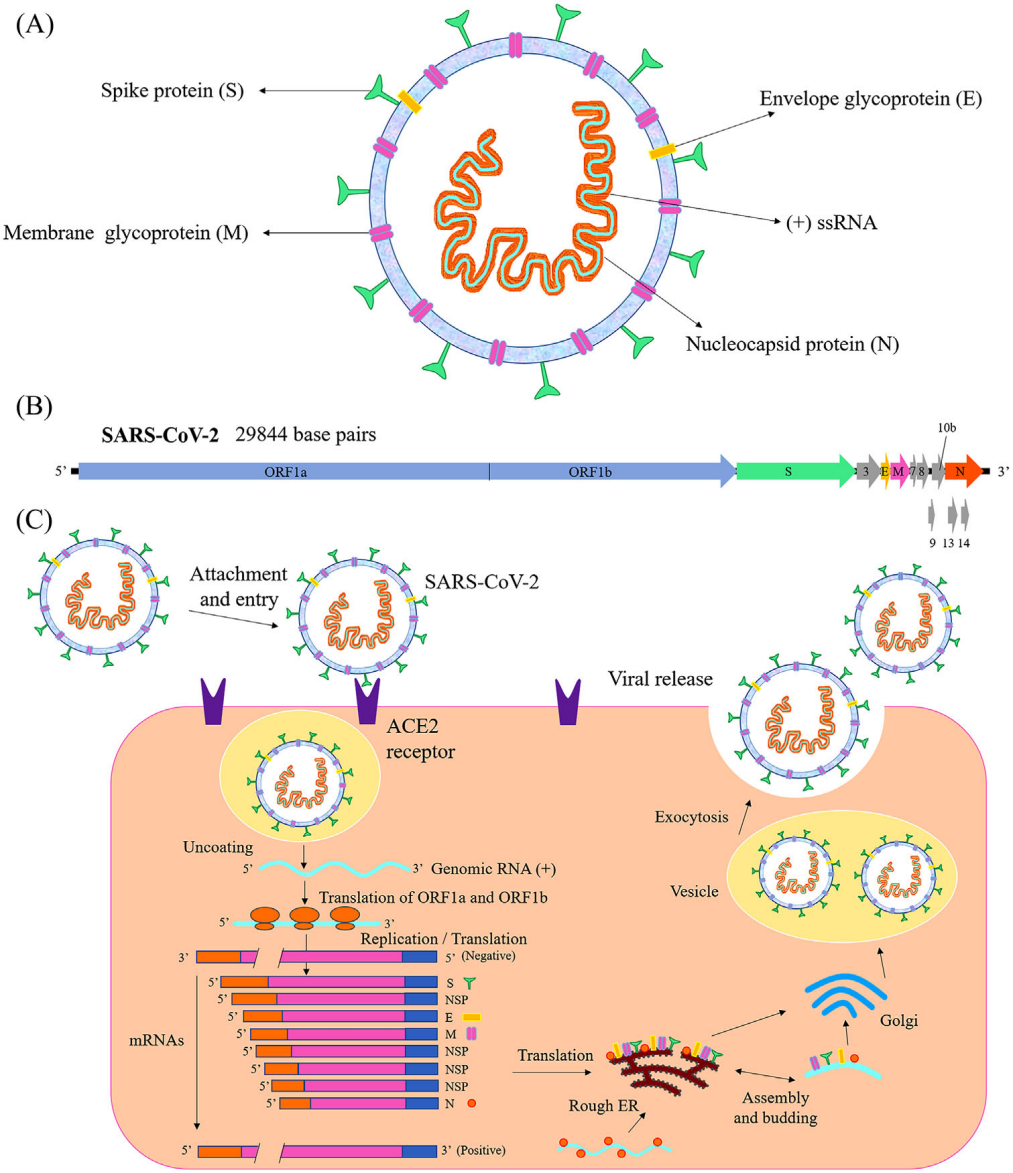
Schematic of the SARS‐CoV‐2 structure (A) and genome (B), the life cycle of SARS‐CoV‐2 in the host cell (C). Reproduced with permission from Shereen et al.^2^ (2020), Copyright 2020 Elsevier. ACE2, angiotensin‐converting enzyme 2; ER, endoplasmic reticulum; mRNA, messenger RNA; ORF, open reading frame; RNA, ribonucleic acid; SARS‐CoV‐2, severe acute respiratory syndrome‐coronaviruses‐2; ssRNA, single‐stranded RNA.

SARS‐CoV‐2 is a spherical enveloped particle involving ssRNA—positive sense—associated with a nucleoprotein within a capsid that consists of matrix protein. The surface of the envelope is covered by spikes with a glycoprotein structure. Also, some CoVs involve a hemagglutinin‐esterase protein. CoVs guanine+cytosine contents change from 32% to 43%.^16^ This virus, which shows the typical β coronavirus organization, has nonstructural proteins (nsp) and structural proteins. The replication enzyme coding region encodes open reading frames (ORFs): ORF1a and ORF1b genes. All CoVs include specific genes in ORF1 downstream regions that encode proteins for nucleocapsid, spikes formation, and viral replication. Sixteen nsp, which are highly conserved throughout the coronavirus and have proceeded from pp1a and pp1b, are encoded by ORF1a/b at the 5′ end, while envelope protein (E), spike protein (S), nucleocapsid protein (N), and membrane protein (M) are encoded by ORFs at the 3′ end^16^, ^17^, ^18^, ^19^, ^20^ as demonstrated in Figure 1A,B. The envelope involves three proteins: M, E, and S. The M protein binds the nucleocapsid (nucleoprotein core) to host membranes and increases budding and viral assembly. The E proteins play multiple roles during infection, including pathogenesis, release, and viral morphogenesis. The S protein is a homotrimer on the virus surface that recognize the cell receptor and is essential for infectivity.^1^

MERS‐coronavirus utilizes dipeptidyl peptidase 4 as a key receptor, while SARS‐coronavirus needs the angiotensin‐converting enzyme 2 (ACE2) to enter the host cell.^21^ The S protein found on the surface of the SARS‐CoV‐2 virus involves a receptor‐binding domain (RBD), similar to SARS‐CoV, which binds to the ACE2 receptor on the host cell for penetrating virus^2^, ^22^ as demonstrated in Figure 1C. After that, the virus reveals its RNA, translates its RNA replicase, and then constructs the RNA replicase–transcriptase complex. This complex creates RNA‐negative strands, which will later be translated into relevant viral proteins via replication and transcription.^19^ Afterward, the RNA and structural proteins are assembled into virions in the rough endoplasmic reticulum and Golgi, then transported by vesicles and finally released outside the infected cells through exocytosis in an attempt to infect other cells.^2^ As a result, one infected cell can release thousands of new viral particles. These particles spread to the bronchi, ultimately achieving the alveoli and extrapulmonary organs, and may cause pneumonia.^19^

## 5 PROTECTION STRATEGIES AGAINST COVID‐19

The most common symptoms of COVID‐19 are dry cough, fever, tiredness, and loss of smell and taste. The less common symptoms are diarrhea, sore throat, headache, pains and aches, irritated or red eyes, and a skin rash or discoloration of toes or fingers. If people have any of these symptoms or have tested positive, they are advised to isolate, take care of themselves, and protect others. In addition, they must stay in touch with their doctor and seek emergency medical care if needed. After isolation period, they can return to their routine.^23^

According to WHO, the management of COVID‐19 has focused chiefly on the prevention of infection, detection and monitoring of cases, and supportive care.^24^ WHO listed simple protective preventions against the COVID‐19 as follows: (i) washing hands with water and soap for at least 40 s, (ii) using disinfectant or cologne including alcohol, (iii) stop rubbing your mouth, nose, and eyes, (iv) keeping the distance at least 1 m with the other person, and (v) using the mask. The person not in a particular risk group can wear a fabric mask, while the risk group should wear a medical/surgical mask. The mask's utilization and disposal after the first usage are crucial.^23^ Mild and moderate COVID‐19 patients and asymptomatic cases should isolate themselves in a well‐ventilated room. If the caregiver enters the room, the patient and the caregiver may consider using an N95 mask. The patients should drink fluids to maintain adequate hydration and rest.^25^

SARS‐CoV‐2 infects various cells in the human body including intestinal cells, lung cells, vascular cells, olfactory epithelial cells, and so forth. The damage caused by the infections of these cells, as well as enduring immune response, lead to long COVID.^26^ Indeed, the crucial and essential protection strategy against the COVID‐19 is developing effective and rapid diagnostic tests and vaccine studies. Several molecular and serological assays have been designed to determine the presence of SARS‐CoV‐2.^27^ Besides diagnostic tests, numerous vaccine and drug development studies have been carried out, and some have even been approved for use. Different strategies, including immunotherapy, cellular therapy, and antiviral therapy, have been applied to treat COVID‐19. Various bioinformatics strategies have also been utilized in this process.^28^

## 6 DIAGNOSTIC TESTS FOR COVID‐19 DETECTION

Early diagnosis is a critical step that allows timely intervention for patients suffering from COVID‐19. More complicated diagnostic tests have become significant in revealing the presence of the SARS‐CoV‐2 virus. Nowadays, commercially produced COVID‐19 tests are classified into two main groups. The first group involves molecular assay techniques based on the polymerase chain reaction (PCR) or strategies related to nucleic acid hybridization to detect SARS‐CoV‐2 viral RNA.^29^ These assays, which use the virus's genetic material, are far more sensitive than other available assays and allow to identify the virus much earlier in clinical samples.^30^

**FIGURE 2:**
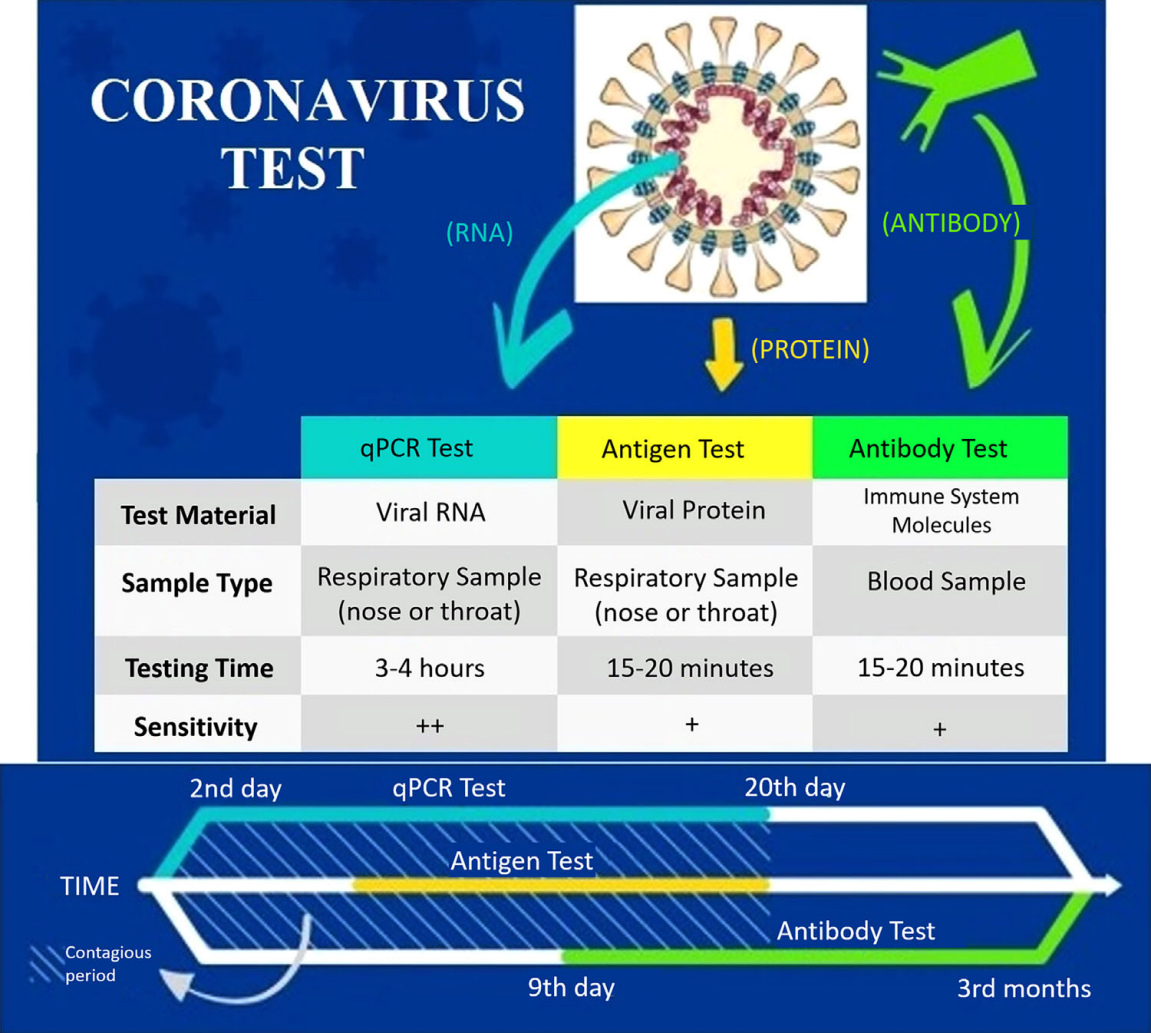
The comparison of COVID‐19 tests. PCR, polymerase chain reaction; RNA, ribonucleic acid.

On the other hand, false‐negative results may occur because of improper collection and handling of the sample or improper extraction of nucleic acid from the sample. Also, false‐negative results are possible due to insufficient amount of virus molecules, cross‐contamination, or inhibitors of amplification.^27^ Due to their high accuracy rate and sensitivity (≈ 100%) in the early stages (between 2 and 3 days to 20 days after exposure), PCR‐based methods are used primarily in hospitals (Figure 2). Moreover, these tests cannot be used at home due to the need for special tools, equipment, laboratories, and specialist personnel.^27^, ^31^, ^32^

The second group involves serological and immunological analyses based on the identification of either antibodies generated by the immune system of infected people or the detection of the antigenic protein^29^ (Figure 2). Both past and current SARS‐CoV‐2 infections can be detected using serological methods. In addition, the progress of the disease periods and immune response could be monitored using these assays. Serological methods are cheaper and take less time to diagnose, and the steps needed to perform the tests are less complicated.^30^ However, there are also some disadvantages: They cannot be used in the early stages of the infection (up to the sixth day) and do not provide information about the viral load. They can make the diagnosis in the later stages of the disease (from the ninth day to the third month).^33^ In addition, the reliability rate of serological kits is limited (89%–99%) due to false‐positive results in patients infected with other human coronavirus types such as HCoV‐OC43 and HCoV‐229E because of cross‐reactivity and false‐negative results in immunocompromised individuals including congenital patients, chronic patients, cancer patients, and elderly patients.^34^ That is why they are not preferred as the primary test in hospitals but allow individuals to test themselves based on the presence–absence of the virus at home.

### 6.1 Molecular assays for diagnosis of SARS‐CoV‐2

In recent years, progress in molecular technology has made these methods one of the most widely used approaches for laboratory diagnosis. It has allowed a better understanding of the epidemiology and pathogenesis of infectious diseases. The virus can be identified and diagnosed in molecular assays by evaluating the genome. Changes in the expression of those genes can be detected over time, especially after the infection. There are three main issues in molecular diagnosis: (i) a high capacity to test large numbers of samples accurately and quickly, (ii) decreasing the number of false‐negative results through detecting small amounts of viral RNA, (iii) preventing false‐positive results via the correct differentiation of positive signals between different pathogens.^35^ Molecular assays such as reverse transcription‐PCR (RT‐PCR), RT loop‐mediated isothermal amplification (RT‐LAMP), clustered regularly interspaced short palindromic repeats (CRISPR), amplicon‐based metagenomic sequencing, and nucleic acid hybridization using microarray provide the accurate diagnosis for SARS‐CoV‐2.^36^

#### 6.1.1 Detection with RT‐PCR

RT‐PCR is based on its potential to amplify minimal amounts of the virus's genetic material in a sample and is regarded as a gold standard for identifying the SARS‐CoV‐2 virus. In this method, it is possible to analyze the samples of potential SARS‐CoV‐2 patients picked up from the feces, blood, and the lower or upper respiratory tract. Swabs have been usually used to obtain specimens from the upper respiratory system in the RT‐PCR COVID‐19 test.^29^ Generally, two sequence regions (N and ORF1b) are selected from Genbank for primer and probe designs that enable in vitro identification of new coronavirus infection with RT‐PCR. The N gene‐based assay is about 10 times more sensitive than the ORF1b gene‐based assay to detect the positive sample. The actual PCR methods have high specificity; however, it is possible to obtain false‐positive results.^1^ Therefore, this system used as a molecular diagnostic test by clinical laboratories needs improvement. The WHO recommended that at least two distinct targets on the SARS‐CoV‐2 virus genome be detected.^37^

RT‐PCR assay begins with the extraction and conversion of viral RNA into deoxyribonucleic acid (DNA) using RNA‐dependent DNA polymerase, also called reverse transcriptase. RT‐PCR reaction is based on the primers, specifically designed for complementary sequences on the virus's genetic material, and reverse transcriptase enzyme in addition to deoxynucleotide triphosphates and cofactor to produce complementary DNA (cDNA) of the viral RNA as shown in Figure S1. In real‐time RT‐PCR, DNA amplification can be monitored using a sequence‐specific probe labeled with a fluorescent dye or a fluorescent molecule. In the automated system, the amplification step is repeated for approximately 40 cycles until the viral cDNA is detected.^29^ Table 2 includes some of the market's RT‐PCR diagnostic tests used to detect SARS‐CoV‐2.

**TABLE 2:** Molecular and serological assays used for SARS‐CoV‐2 diagnosis.

**Test Name**	**Manufacturer name**	**Sample source**	**Detected region**	**Total result time**	**Ref**.
**Real‐time reverse transcription‐polymerase chain reaction**					
CDC 2019‐Novel Coronavirus Real‐Time RT‐PCR Diagnostic Panel	CDC	Sputum, nasal aspirate, NS, OS	N gene	–	^67^
Simplexa COVID‐19 Direct Kit	DiaSorin Molecular	NS	ORF1ab and S gene	~ 1 h	^68^
COVID‐19 RT‐PCR Test	Laboratory Corporation of America	Sputum, nasal aspirate, NS, OS	N gene	48–96 h	^69^
ARIES SARS‐CoV‐2 Assay	Luminex Corporation	NS	ORF1ab and N gene	~ 2 h	^70^
Novel Coronavirus (2019‐nCoV) Nucleic Acid Diagnostic Kit	Sansure Biotech Inc.	NS, OS, alveolar lavage fluid, sputum, whole blood, feces	ORF1ab and N genes	30 min	^71^
COVID‐19 Coronavirus Real Time PCR Kit	Eagle Biosciences	NS, OS, anterior nasal swabs, mid‐turbinate nasal swabs, nasal aspirates, nasal washes, BAL fluid, sputum	ORF1ab and N genes	72 min	^72^
COVID‐19 RT‐PCR PNA Kit	BioTNS	NS, OS, anterior nasal swabs, mid‐turbinate nasal swabs, BAL, nasopharyngeal wash/aspirates, nasal aspirates specimens	N and RdRP genes	140 min	^73^
STANDARD M nCoV Real‐Time Detection Kit	SD Biosensor Inc.	NS, OS, nasal mid‐turbinate, anterior nares specimen, sputum	N and RdRP genes	90 min	^74^
Bio‐Speedy Direct RT‐qPCR SARS‐CoV‐2	Bioeksen R&D Technologies Inc.	NS, OS, BAL, nasopharyngeal aspirate, sputum, saliva, gargle	ORF1ab	65 min	^75^
**RT‐LAMP**					
ID NOW COVID‐19	Abbot Diagnostics Scarborough, Inc.	Nasal, throat, NS	RdRp gene	5–13 min	^76^
Isopollo COVID‐19 Detection Kit	M Monitor Inc.	NS, OS, sputum, BAL	N and RdRP genes	30 min	^77^
Loopamp™ SARS‐CoV‐2 Detection Kit	Eiken Chemical Co. Ltd.	NS, OS, saliva	N and RdRP genes	60 min	^78^
MobileDetect Bio BCC19 Test Kit	Detectachem Inc.	Nasal swab, NS, OS	E and N genes	30 min	^79^
Detect COVID‐19 Test	Detect Inc.	Anterior nasal swabs	ORF1ab	1 h	^80^
Lucira™ COVID‐19 All‐In‐One Test Kit	Lucira Health	Nasal swab	N gene	30 min	^81^
**Clustered regularly interspaced short palindromic repeats (CRISPR)**					
SARS‐CoV‐2 DETECTR	Mammoth Biosciences	Respiratory samples	E and N genes	30–40 min	^82^
Sherlock CRISPR SARS‐CoV‐2 Kit	Sherlock Biosciences	Anterior nasal swabs, NS, OS, nasopharyngeal wash/aspirate, nasal aspirate, BAL	ORF1ab, N gene	~ 1 h	^83^
TATA MD CHECK CRISPR SARS‐COV‐2 KIT 1.0	Tata Medical and Diagnostics	NS, OS	S gene	1.67 h	^84^
**Amplicon‐based metagenomic sequencing**					
ISARIC 4C (scientific work)	ISARIC 4C Consortium	NS	ORF1ab	8 h	^43^
**Nucleic acid hybridization using microarray + RT‐PCR**					
Detect^X^‐Rv	PathogenDx, Inc.	NS, OS, mid‐turbinate nasal swabs, anterior nasal swabs, nasal aspirates, nasopharyngeal wash/aspirates, BAL	N1, N2 genes	–	^85^
CovidArray	Italy (scientific work)	NS	N1, N2 genes	125 min	^86^
**Enzyme‐linked immunosorbent assay (ELISA)**					
SARS‐CoV‐2 IgG ELISA Kit	Creative Diagnostics	Serum, plasma	IgG	–	^87^
EDI Novel Coronavirus COVID‐19 IgM ELISA Kit	Epitope Diagnostics, Inc.	Serum, plasma	IgM	120 min	^88^
AMP ELISA TEST SARS‐COV‐2 AB	AMEDA Labordiagnostik GmbH	Serum, plasma	Total Antibody	70 min	^89^
Anti‐SARS‐CoV‐2 QuantiVac ELISA (IgG)	EUROIMMUN AG	Fingerprick blood, serum, plasma	IgG	120 min	^90^
GB SARS‐CoV‐2 Ab ELISA	General Biologicals Corporation	Serum, plasma	Antibody	120 min	^91^
**Lateral flow test**					
COVID‐19 IgM/IgG Rapid Test	BioMedomics Inc.	Fingerprick or whole blood, serum, plasma samples	IgG/IgM	10 min	^51^
COVID‐19 Ag Respi‐Strip	Coris Bioconcept	NS	N protein antigen	15–30 min	^92^
CRT COVID‐19 Rapid Test	Oncosem Onkolojik Sistemler San. ve Tic. A.S.	Blood, plasma, serum	IgG/IgM	10–15 min	^93^
Rapid COVID‐19 IgM/IgG Combo Test Kit	Megna Health Inc.	Serum, ACD plasma, fingerstick whole blood	IgG/IgM	15 min	^94^
Pilot™ COVID‐19 At‐Home Test	SD Biosensor Inc.	Anterior nasal swabs	N protein antigen	20 min	^95^
INDICAID COVID‐19 Rapid Antigen At‐Home Test	PHASE Scientific International, Ltd.	Nasal swabs, NS	N protein antigen	20 min	^96^
VivaDiag™ SARS‐CoV‐2 IgM/IgG Rapid Test	VivaChek Biotech Co., Ltd.	Plasma, serum, whole blood	IgG/IgM	15 min	^97^
SCoV‐2 Ag Detect Rapid Self‐Test	InBios International Inc.	Anterior nasal swabs	N protein antigen	25 min	^98^
BinaxNOW COVID‐19 Ag Card Home Test	Abbott Diagnostics Scarborough, Inc.	Anterior nasal swabs	N protein antigen	15 min	^99^
iHealth COVID‐19 Antigen Rapid Test Pro	iHealth Labs, Inc.	Anterior nasal swabs	N protein antigen	15 min	^100^
SPERA COVID‐19 Ag Test	Xtrava Health	Anterior nasal swabs	N protein antigen	15 min	^101^
NIDS COVID‐19 Antigen Rapid Test Kit	ANP Technologies, Inc.	Mid‐turbinate nasal swabs	N protein antigen	15 min	^102^

Abbreviations: ACD, acid citrate dextrose; BAL, bronchoalveolar lavage; CDC, Centers for Disease Control and Prevention; DETECTR, deoxyribonucleic acid (DNA) endonuclease‐targeted CRISPR trans reporter; IgG, immunoglobulin G; IgM, immunoglobulin M; NS, nasopharyngeal swabs; OS, oropharyngeal swabs; RdRp, ribonucleic acid (RNA)‐dependent RNA polymerase.

#### 6.1.2 RT‐LAMP assay

The LAMP is a nucleic acid amplification method that amplifies DNA under isothermal conditions with high selectivity, specificity, sensitivity, and rapidity, thus eliminating the need for a thermal cycler.^38^ The absence of a thermal cycler reduces the diagnostic device's complexity and cost.^39^ The amplification efficiency of this method is very high, and due to its isothermal reaction, there is no time loss for thermal change.^38^ In order to increase the sensitivity, RT‐LAMP requires a set of 4–6 primers specific for 6–8 different target DNA regions and combines LAMP with the RT step as demonstrated in Figure S2.^40^ A strand‐displacing DNA polymerase initiates synthesis. Also, two primers generate loop structures to facilitate subsequent amplification steps. As a byproduct of amplification, magnesium pyrophosphate precipitate forms in the solution, and this precipitate causes turbidity that can be detected by photometry. Also, this reaction could be followed in real time by fluorescence using intercalating dyes and measuring turbidity. Since the RT‐LAMP diagnostic test requires only real‐time heating and visual inspection, the sensitivity and simplicity of this tool make it a hopeful candidate for virus detection.^29^ However, this method has some difficulties, such as optimizing reaction conditions and designing sequence‐specific primers. It is possible to overcome these drawbacks using the CRISPR method.^39^ Table 2 includes some of the RT‐LAMP assays used to detect SARS‐CoV‐2 in the market.

#### 6.1.3 Assay based on CRISPR

CRISPR, one of the effective gene‐editing methods, performs the recognition and cutting of targets in DNA or RNA sequences using bacterial enzymes such as Cas12 or Cas13. This method is used not only as a gene‐editing tool but also as a molecular diagnostic tool for detecting infectious diseases, including COVID‐19. CRISPR has high sensitivity and high selectivity and offers fast and specific read‐out. It could be run several times on the same sample, reducing the possibility of false‐negative results. DNA endonuclease‐targeted CRISPR trans reporter (DETECTR) technique and the specific high sensitivity enzymatic reporter unlocking (SHERLOCK) technique are two different CRISPR‐based assays used to detect viral RNA.^39^

SHERLOCK technique based on CRISPR can ultra‐sensitively detect RNA or DNA, as demonstrated in Figure S3. This method uses Cas13, which can excise reporter RNA sequences in response to activation via the SARS‐CoV‐2‐specific guide RNA. Viral RNA is converted to double‐stranded DNA (dsDNA) by recombinase polymerase amplification (RT‐RPA), and then complementary RNA is generated from the dsDNA template with T7 transcription. The Cas13‐tracrRNA (i.e., trans‐activating CRISPR RNA) complex binds to the target sequence, and thus the nuclease enzyme activity of Cas13 is activated to cleave the target sequence and the fluorescent RNA reporter. The DETECTR technique allows cleavage of the reporter RNA via Cas12a for specific detection of viral RNA sequences—the N and E genes. After that, the isothermal amplification of the target takes place and finally leads to a visual read‐out with a fluorophore. These tests have a low cost and short response time (almost 1 h), which is very important for point‐of‐care (PoC) diagnosis.^29^, ^41^ Table 2 includes some assays based on the CRISPR used to detect SARS‐CoV‐2.

#### 6.1.4 Detection with amplicon‐based metagenomic sequencing

Amplicon sequencing is a highly targeted technique that enables genetic variation to be analyzed in specific genomic regions. The ultra‐deep sequencing of PCR products (amplicons) provides efficient variant identification and characterization. This technique helps detect complex samples of uncommon somatic mutations. It enables researchers to sequence targets ranging from a few to 100 genes in a single process.^42^ On the other hand, the metagenomic sequencing technique is used to tackle the background microbiome of those infected.^43^ This approach allows the rapid detection of the SARS‐CoV‐2 virus and other pathogens that lead to secondary infections. In addition, metagenomic methods such as sequence‐independent single primer amplification ensure additional control on sequence divergence. This dual technique can evaluate the mutation rate and identify possible recombination with other human CoVs. These issues are crucial regarding antiviral efficacy and vaccine development.^29^ Nanopore sequencing has excellent potential as an emerging technology that allows a single DNA or RNA molecule to be sequenced in real‐time. The MinION, the first commercial sequencer based on the nanopore technology released by Oxford Nanopore Technologies, is a small and powerful sequencing instrument suitable for the genetic analysis of pathogens.^44^ Amplicon‐based MinION sequencing has been used to rapidly (∼8 h) sequence the SARS‐CoV‐2 genome and the nasopharyngeal swab microbiome collected from COVID‐19 patients^29^, ^43^ (Table 2).

#### 6.1.5 Nucleic acid hybridization using microarray

Microarray is a highly efficient diagnostic method used to detect SARS‐CoV‐2 nucleic acids. This method produces cDNAs from viral RNA and reference RNA via RT and then labeled with specific probes. In the next step, viral cDNA and reference cDNA, both of which are with different fluorescent labels, are mixed. These labeled cDNAs are hybridized with solid‐phase oligonucleotides fixed on the microarray by loading into each well as demonstrated in Figure S4.^29^ After a series of washing steps to remove the free DNA, coronavirus RNA can be detected by following the specific probes.^45^ In the literature, this technology has detected various single nucleotide polymorphisms in the S gene of SARS‐CoV‐2 with 100% accuracy.^46^ Table 2 includes some of the microarrays that are used to detect SARS‐CoV‐2.

### 6.2 Serological assays for diagnosis of SARS‐CoV‐2

Serological assays have been used to test the existence of immunoglobulin G (IgG) and immunoglobulin M (IgM) and antibodies produced by the immune system. They detect the presence of antibodies to the virus in the patient's blood, serum, saliva, plasma, sputum, and other biological fluids instead of detecting viral antigens.^39^, ^47^ Serological tests play a significant role in the development of vaccines and epidemiology, offering an evaluation of both long‐term (years or permanence) and short‐term (days to weeks) trajectories of antibody response, abundance, and diversity.^29^ Following the SARS‐CoV‐2 infection, IgM becomes detectable in serum after a few days, peaks within 2 weeks, and is reduced to near‐background levels. On the other hand, IgG is produced after a week, peaks 3 weeks, and is maintained at a high level for an extended period. The serological tests can be used as a confirmative method in detecting SARS‐CoV‐2 to check the accuracy of the nucleic acid tests.^29^, ^48^ These tests, such as detection with enzyme‐linked immunosorbent assay (ELISA) and detection with lateral flow test, provide a rapid diagnosis for SARS‐CoV‐2 infections.

#### 6.2.1 Detection with ELISA

ELISA is a plate‐based assay designed to identify and quantify substances such as antibodies, peptides, proteins, and hormones within typically 1−5 h.^29^ This assay, which could be quantitative or qualitative, is quite sensitive to IgG from the 10 days following the initial symptoms. Therefore, the ELISA method, targeting the nucleocapsid or S protein of the virus, has been used for SARS‐CoV‐2 specific antibody responses and the identification of infection.^49^, ^50^ In this method, the plate wells are coated with a viral protein to ensure specific binding of antiviral antibodies in the samples of patients if they exist. Then secondary enzyme‐labeled antibody binds to the patient's antibody. Finally, a colorimetric or fluorescent‐based read‐out can be obtained as a result of enzymatic conversion of the substrate as demonstrated in Figure S5.^29^ Table 2 includes some of the ELISA assays used to detect SARS‐CoV‐2 in the market.

#### 6.2.2 Detection with lateral flow test

Lateral flow test is a qualitative chromatographic assay, which is portable, small, and used for PoC testing. This cheap test does not require trained personnel and gives qualitative results. This test is a kind of rapid diagnostic test because of getting results in 10 to 30 min. Lateral flow tests can detect SARS‐CoV‐2 viral antigens, including the S protein or N protein. In this assay, liquid samples such as blood, plasma, and serum are applied to a substrate, allowing the sample to flow through a band of immobilized viral antigens. If they exist, anti‐SARS‐CoV‐2 antibodies are gathered at the band, where, along with co‐collected tracer antibodies, a color develops to demonstrate to test result. A diagnosis of infection could be possible when used in combination with symptomology.^29^ Various companies developed and launched rapid PoC lateral immunoassays to diagnose coronavirus infection. One of these tests has been developed by BioMedomics Inc., which can detect early and late markers in serum, plasma, whole venous blood, or fingerprick. The test card includes colloidal gold‐labeled recombinant novel coronavirus antigen, two detection lines—M line and G line—and one quality control line—C line—fixed on a nitrocellulose membrane. When an adequate quantity of the test material is inserted into the sample well, it will move forward along with the test card through capillary action. The antibody will bind to the colloidal gold‐labeled recombinant coronavirus antigen if the sample includes IgM. The antigen/antibody complex will be captured via the antihuman IgM antibody, immobilized on the membrane, generating a red M line that suggests a positive outcome for the IgM antibody. Likewise, the same process will perform with IgG antibodies. A negative result is indicated if no antibodies are present.^51^ This workflow is shown in Figure S6. Table 2 includes some lateral flow tests used to detect SARS‐CoV‐2 in the market.

### 6.3 Comparison of accuracy rate and cost of different detection assays

Assays used in the diagnosis of COVID‐19 have different advantages and disadvantages. RT‐PCR techniques are costly, that is why many countries—especially low‐income countries—cannot afford enough number of tests in order to screen a large population.^52^ The assay time can vary between 30–120 min for RT‐PCR. The sensitivity and specificity are high.^53^ According to a recent meta‐analysis, the sensitivity of RT‐PCR for the detection of SARS‐CoV‐2 in sputum and NS samples was 97.2% and 73.3%, respectively.^54^ Also RT‐LAMP has high sensitivity and specificity.^52^ According to a systematic review, the sensitivity of RT‐LAMP was 78% in crude samples and 94% in purified RNA from samples of COVID‐19 patients. Also, RT‐LAMP was susceptible to producing false‐negative results in samples with low viral loads.^55^ This assay is rapid and cost‐effective. According to a study result, when all related costs are included, the average per‐test cost for the real‐time RT‐PCR was $14.75 and for RT‐LAMP was $8.45.^56^ The sensitivity and specificity of CRISPR‐based assays are high.^53^ SHERLOCK for SARS‐CoV‐2 detection has demonstrated 93.1% sensitivity and is significantly faster than qRT‐PCR.^57^ It was reported that depending on the assay technique, CRISPR‐COVID assay costs less than $3.50 for a single reaction.^58^ In the near future, CRISPR‐based assays have the potential to become less complicated, more reliable, faster, and more affordable.^59^ The detection time is 2–8 h for ELISA. The sensitivity is low and specificity is intermediate.^53^ Many ELISA kits have been developed during the COVID‐19 pandemic. The sensitivity range can be from 84% to 94% and the specificity can be up to 100%.^60^, ^61^, ^62^ Costs of these tests range from $445 to $785 for 96 tests.^63^ Rapid antigen tests are relatively inexpensive and give results within 10–15 min.^64^ But the accuracy of rapid antigen tests is lower than standard RT‐PCR tests.^65^ According to a meta‐analysis including 17,171 suspected COVID‐19 patients, sensitivity and specificity of rapid antigen test kits were detected as 68.4% and 99.4%, respectively.^66^ For the early detection of patients suspected of having COVID‐19, the use of rapid antigen test kits is often advised, especially in remote, densely populated areas with few resources and laboratory equipment.

## 7 VACCINE STUDIES FOR COVID‐19 DISEASE

### 7.1 Vaccine studies against viral infections

Four major platforms have been investigated for the SARS‐CoV‐2 vaccines: protein‐based, viral‐vector, whole virus, and nucleic acid vaccines.^103^, ^104^ The contents, advantages, disadvantages, storage conditions, and side effects of these vaccine platforms are summarized in Table 3.^103^, ^105^, ^106^, ^107^

**TABLE 3:** Comparison of four vaccine types.

**Vaccine platform**	**Contains**	**Advantages**	**Disadvantages**	**Storage**	**Side effects**
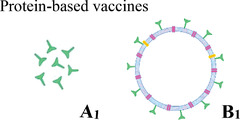	Isolated and purified viral proteins **(A_1_: subunit)** or viral proteins, which mimic the structure of the virus, but no genetic material **(B_1_: virus‐like particle)**	Well‐established technology, no risk of triggering the disease, broad antigenic profile	Complex to manufacture, poorly immunogenic	2–8°C	Low
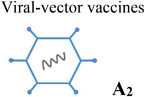	Viral genetic material packaged inside another harmless virus, which can copy itself **(replicating viral vector)** or cannot copy itself **(nonreplicating viral vector)**	Well‐established technology, strong immune response	Complex to manufacture, pre‐existing immunity against the vector could reduce the effectiveness	2–8°C	Pain at the injection site, joint pain, headaches, fatigue, chills, muscle pain, fever
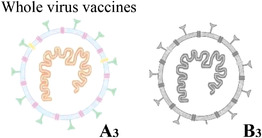	Copies of the virus that have been killed **(A_3_: attenuated)** or weakened **(B_3_: inactivated)**	Well established technology, simple to manufacture, broad antigenic profile	Live attenuated vaccine may trigger disease in very rare cases	2–8°C	Elevated blood pressure, injection site pain, headache, rash, dizziness
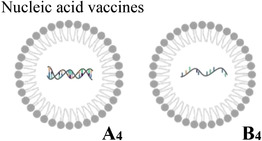	Viral genetic material **(A_4_: deoxyribonucleic acid [DNA]‐based or B_4_: ribonucleic acid [RNA]‐based)** that provides the instructions for making viral proteins	Strong immune response, easy production, no risk of the vaccine triggering disease	Need ultra‐cold storage and special delivery systems	−70°C	Tiredness, headache, muscle pain, chills, fever, nausea

#### 7.1.1 Protein‐based vaccines

Protein‐based vaccines could be divided into two groups that are subunit vaccines and virus‐like particle (VLP) vaccines.^103^ Protein subunit vaccines, which are produced through recombinant protein strategies, are generated easily. Also, they are relatively well‐tolerated and safe, compared to whole virus vaccines. Adjuvants are generally used with these vaccines because of their low immunogenicity. Moreover, the adjuvant could be optimized, and booster doses could be increased to provide a more robust and consistent level of immunity.^103^, ^108^ The chances of unfavorable reactions to the vaccine are lower because subunit vaccines comprise only the essential part of the antigens and not the whole virus.^109^ Thus, concerns such as virulence recovery, pre‐existing antivector immunity, and incomplete viral inactivation could be eliminated.^110^ They may include variable antigens ranging from 1 to 20 antigens (Table 3, A_1_). The subunit vaccines have been produced for hepatitis B and *Haemophilus influenza* A and B.^111^

On the other hand, VLP vaccines use empty virus shells, which mimic the structure of the virus (Table 3, B_1_), and induce a robust immune response against the antigen presented on its surface. These vaccines are not infectious because they lack genetic material.^103^, ^108^ VLPs mimic virions morphologically and immunologically, so they could induce high titers of neutralizing antibodies (nAbs) to conformational epitopes. They can induce an immune response as if the immune system has been exposed to an actual virus.^109^ VLPs, which fall in the size range of viruses (generally 22–200 nm), are unable to replicate in the recipient; however, they can stimulate the immune system via recognition of repetitive subunits, generating high humoral and cellular immune responses.^112^, ^113^

Moreover, their repetitive, high‐density display of epitopes is potentially highly effective in enhanced immunogenicity responses.^112^ VLP vaccines can be made by recombinant expression and allow for the incorporation of ligands, targeting moieties, and immunomodulators.^114^ They are excellent prophylactics and eliminate virus‐based vaccine limitations.^109^ VLPs offer a promising approach to produce vaccines against many diseases, including Ebola, influenza A, Zika, and respiratory syncytial virus infections.^115^

#### 7.1.2 Viral‐vector vaccines

Viral‐vector vaccines, whose concept is different from that of subunit vaccines, could combine numerous properties of DNA vaccines with live attenuated vaccines.^116^, ^117^ The main advantage of this vaccine is that the vector alone can achieve the goal of eliciting a protective immune response, so it may not need an adjuvant.^118^ The viral vector acts as a delivery system that invades the cell, inserts the code for various viral antigens, and finally transduces cells to create the desired immunized result.^119^ The virus used as a vector, including the adenovirus, vaccinia virus, and measles virus, is chemically weakened. That is why it cannot cause the disease.^103^ The efficacy and safety of viral‐vector vaccines need to be evaluated using different assays involving genetic stability, immunogenicity, genotoxicity, replication deficiency, or attenuation. As infectious diseases are one of the significant problems in developing countries, it is crucial to enable large‐scale manufacturing of viral vectors.^116^ Viral‐vector vaccines are divided into two groups that are replicative and nonreplicative vector‐based vaccines where key genes have been disabled. These vaccines are produced by integrating exogenous protective antigen‐encoding genes into the viral genome whose deleterious genes have been removed (Table 3, A_2_).^10^, ^103^ These vaccines appear to be safe and provoke a robust immune response.

On the other hand, if a person has been exposed to the viral vector and has an improved immune response, this situation could blunt the vaccine's effectiveness.^103^, ^120^ Although different types of viral vectors have been developed, only one vaccine had been approved in humans against Ebola virus disease, a fatal illness, before the onset of SARS‐CoV‐2.^103^, ^121^ The Ebola vaccine uses the vesicular stomatitis virus as a replicative viral vector, which has little or no effect on humans.^103^, ^122^

#### 7.1.3 Whole virus vaccines

Whole virus vaccines use an attenuated or inactivated form of the virus to induce protective immunity.^103^ An essential advantage of these vaccines is their inherent immunogenicity and ability to stimulate toll‐like receptors (TLRs), including TLR3, TLR7/8, and TLR9.^123^ Live attenuated vaccines utilize a weakened form of the virus that can still replicate and grow; however, they are almost or entirely devoid of pathogenicity (Table 3, A_3_). They do not cause disease but can induce a protective immune response against various virus antigens. The other advantage of these vaccines is the possibility for scale‐up for mass production.^103^, ^110^, ^111^ These vaccines are like natural infections and can have a long‐lasting effect. Most live vaccines only require one or two doses to achieve the desired immune response. However, live vaccines require extended safety and extra caution for people with long‐term health problems, weakened immune systems, or organ transplants.

Moreover, they should be well preserved in cold storage.^109^ Another disadvantage of these vaccines is that secondary mutations may cause the reversion of virulence and finally lead to disease.^111^ Live attenuated vaccines against different viruses, including poliovirus, influenza, measles, rotavirus, and yellow fever virus, have been commercialized.^110^

Inactivated vaccines comprise viruses whose genetic material has been destroyed using chemicals such as formaldehyde or β‐propiolactane and/or physical methods such as heat or radiation (Table 3, B_3_). Therefore, they cannot infect the cells and replicate; however, they can trigger an immune response.^103^, ^111^ Inactivated vaccines generally do not provide immunity as strong as live vaccines, so multiple doses are required. On the other hand, inactivated vaccines are safer and more stable than live vaccines because dead viruses cannot mutate back to their pathogenic state. In addition, they are easy to store and transport because they do not require cold storage.^109^ Also, they do not involve genetic manipulation and have low production costs.^124^ Many approved vaccines are inactivated, including influenza, polio, cholera, typhoid, pertussis, and plague.^110^

#### 7.1.4 Nucleic acid vaccines

Nucleic acid vaccines utilize genetic instructions, in the form of DNA or RNA (ribonucleic acid), as depicted in Table 3 A_4_, for a protein that prompts an immune response.^103^ DNA or RNA vaccines indicate CD8+ cytotoxic T‐cell responses that play an essential role in eradicating the virus, in addition to antibody and CD4+ T‐cell responses.^114^ DNA vaccines, which have emerged as a promising alternative to traditional protein‐based vaccines, include selected gene/genes of the virus in the form of DNA.^109^, ^110^ They are based on a recombinant eukaryotic expression vector that encodes a particular protein antigen and is directly injected into the organism. Thus, the foreign gene is expressed in vivo, and the antigen induces the immune system.

Furthermore finally, specific cellular and humoral immune responses are stimulated. Adjuvants can also enhance the prophylactic and treatment effects.^10^ These vaccines could not cause disease because they contain only copies of a few virus genes. DNA vaccines offer many advantages, including safety, increased stability, scalability for mass production, rapid and inexpensive production, long‐term persistence of immunogens, and flexibility to generate vaccines for various infectious diseases.^10^, ^109^, ^110^ DNA vaccines can be produced within weeks as no culture or fermentation is required.^124^

On the other hand, these vaccines have some disadvantages. DNA injected into the body is rapidly degraded and may risk autoimmunity.^10^ In addition, there is limited positive clinical data on DNA vaccines in the literature. Unfortunately, no DNA vaccine has been licensed.^110^

RNA vaccines include selected virus genes in the form of mRNA (messenger RNA) as depicted in Table 3, B_4_, and, after cytosolic delivery, these genes are translated into viral proteins.^110^ DNA vaccines need to get into the nucleus, whereas mRNA vaccines only require to get into the cytoplasm to express the target antigen. Therefore, mRNA vaccines are safer than DNA vaccines theoretically. On the other hand, DNA vaccines are considerably more temparature‐stable compared to mRNA vaccines. The immunogenicity, stability, and half‐life of mRNA could be modulated by established modifications.^10^, ^114^ mRNA vaccines can be produced quickly, making the process comparatively easy to monitor.^10^ The RNA could be encapsulated within nanoparticles, injected by itself, or driven into cells through some techniques. When RNA is inside the cell and begins generating antigens, they are displayed on its surface, and the immune response is triggered.^103^ The prophylactic mRNA vaccines could be divided into two main groups: nonreplicating and self‐amplifying RNA vaccines. The nonreplicating mRNA vaccines, also called conventional mRNA vaccines, contain 5′ untranslated region (5′UTR) and 3′UTR that flank the antigenic or immunomodulatory sequence.^10^, ^125^ A poly(A) tail could be added enzymatically after in vitro transcription or incorporated from the 3′ end of the plasmid DNA (pDNA) template.

On the other hand, self‐amplifying RNA vaccine pDNA templates include additional alphavirus replicon genes and conserved sequence elements. Conventional mRNA and synthetic self‐amplifying RNA vaccines are produced in the same way. An mRNA expression plasmid encoding a DNA‐dependent RNA polymerase promoter and the RNA vaccine candidate is designed as a template for in vitro transcription, which is cell‐free. Compared to conventional mRNA, self‐amplifying RNAs have enhanced antigen expression at lower doses. On the other hand, conventional and self‐amplifying mRNA vaccines have demonstrated a protective effect in preclinical trials against various infectious diseases, including Ebola, influenza, Human Immunodeficiency Virus‐1 (HIV‐1), Rabies, and Respiratory Syncytial Virus (RSV).^125^ However, nonreplicating mRNA vaccines have a short RNA sequence and simple structure. Except for the antigen, no additional proteins are required.^10^ One of the disadvantages of mRNA vaccines is that they can cause an anaphylactic reaction.^126^ Also, the storage and transportation of mRNA vaccines need ultra‐low temperatures.^127^

### 7.2 Steps in vaccine development

**FIGURE 3:**
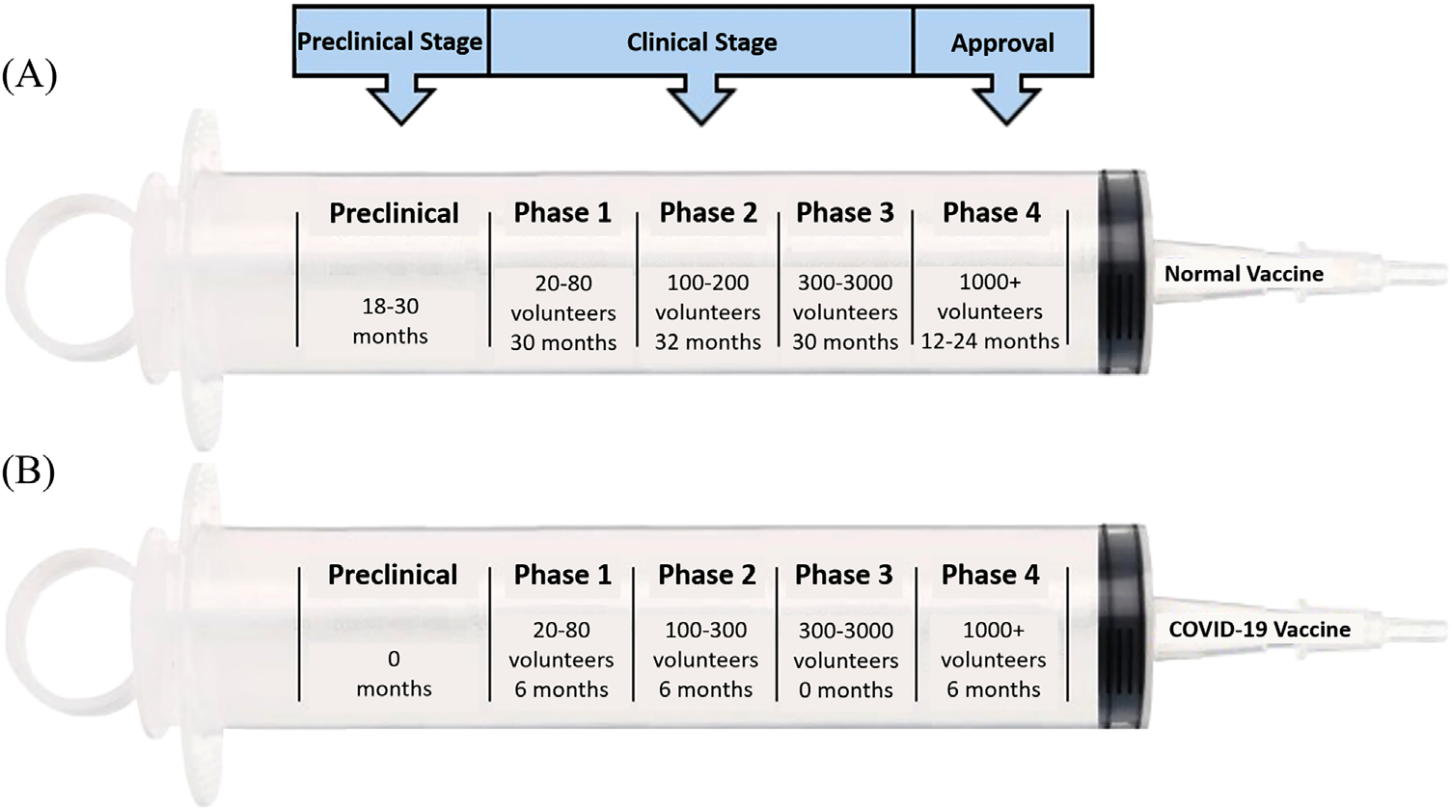
A scheme to compare (A) regular and (B) potential COVID‐19 vaccines.

The first step in the vaccine development process is identifying the vaccine candidate. This stage is called preclinical development, which determines whether the vaccine is effective and safe. First, antigen and suitable technology are selected, and in vitro and in vivo tests are applied.^128^ The second step is the clinical stage, which aims to demonstrate efficacy, immunogenicity, and safety, including phase I, phase II, and phase III vaccine trials. These three phases may overlap, and multiple phase I or phase II trials could be performed. In phase I, the candidate vaccine is evaluated by its application on a limited number of volunteers (usually between 20 and 80) to evaluate its effectiveness, decide the dosage, and classify the side effects. These results are collected by comparing the vaccine with a control or a placebo (e.g., saline), an inactive product. Data on the dosage and the period between vaccinations needed to ensure the optimum response of the immune system are determined herein. Vaccines that pass the first phase are applied to a larger group of volunteers (usually between 100 and 300) in the second phase to determine their safety and immunogenicity. In phase II, which takes at least 2 years, the vaccine's appropriate dosage and administration schedule are investigated in detail. In addition, this phase is well‐controlled and frequently randomized.^128^, ^129^, ^130^ Phase III and phase II designs are similar but in different sizes.^131^ Phase III, where 3000–50,000 volunteers are enrolled, is usually performed with the most promising candidate vaccines. This process, which may take 3–5 years, aims to assess the efficacy of the vaccines in a large‐scale population. In addition, concomitant administration with other vaccines is checked herein. In the next step, these vaccines are approved by regulatory agencies, including the European Medicines Agency in the European Union and Food and Drug Administration (FDA) in the United States. After this stage, the vaccine can be produced on a large scale. The vaccines reached phase IV, also defined as pharmacovigilance, which aims to detect adverse events early and enter the pharmaceutical industry. This phase includes strict monitoring of vaccines in order to identify, evaluate, recognize, prevent, and communicate any adverse effects following immunization or any other aspects relevant to the immunization. Summarily, the development of a vaccine takes more than a decade as depicted in Figure 3A since (i) it is necessary to test on tens of thousands of volunteers, (ii) the safety and efficacy of vaccines have to be regulated, not only by suppliers but also by competent authorities, (iii) requirement to determine whether the vaccine's safety is long‐lasting. However, in order to overcome the pandemic in a short time, scientists around the world have managed to develop COVID‐19 vaccines against the SARS‐CoV‐2 virus bypassing all these stages quickly^128^, ^130^, ^132^ as seen in Figure 3B. So, the development of COVID‐19 vaccines include new concepts such as large production capacity, parallel and adaptive development phases, and innovative regulatory processes. It should not be forgotten that the principles of the 3Rs, which are replacement, reduction, and refinement, must be considered in the vaccine development process.^130^

### 7.3 COVID‐19 vaccine studies

To control the COVID‐19 pandemic, producing effective vaccines is very important. Vaccines could reduce person‐to‐person transmission, disease severity, and viral shedding. There are many promising targets, including the S protein and RBD subunit for SARS‐CoV‐2.^124^ Especially the composition of the S protein, which is a trimeric class I fusion protein, has become significant for COVID‐19 vaccine development because of its essential role in membrane fusion and receptor binding. S protein typically exists in a metastable and prefusion conformation. When the virus interacts with the host cell, the S protein undergoes structural changes, and the viral membrane fuses with the host cell membrane. Characterizing the prefusion S structure at the atomic level guides the design and development of the vaccine for COVID‐19.^110^, ^133^ Nowadays, there are many different vaccine studies in the production process to protect from COVID‐19. As of January 21, 2023, 168,47 vaccine doses have been administered per 100 population.^9^

As of April 8, 2022, WHO has evaluated nine vaccines: Pfizer/BioNTech, Moderna, Johnson and Johnson, AstraZeneca/Oxford, Sinovac, Sinopharm, Covaxin, Covovax, and Nuvaxovid against COVID‐19 that have met the necessary criteria for safety and efficacy.^134^ The immunogenicity of these vaccines characterizes their clinical efficacy. However, these vaccines have common adverse events such as erythema, pain, swelling at the injection site, myalgia, chills, and weakness.^135^

Pfizer/BioNTech (BNT162b2) vaccine is based on mRNA technology, which is delivered in a lipid nanoparticle to express a full‐length SARS‐CoV‐2 S protein.^136^ This vaccine induces both nAbs and cellular immune responses. The administration of this vaccine in two doses 21 days apart gave 95% efficacy in preventing COVID‐19 in subjects.^137^ As requires storage at −80 to −60°C, logistic problems may occur.^138^ It could be stored for 1 month at 2 to 8°C.^139^ Moderna (mRNA‐1273) utilizes the same mRNA technology as Pfizer/BioNTech and uses mRNA delivered in a lipid nanoparticle to express a full‐length SARS‐CoV‐2 S protein. This vaccine has a similarly high efficacy (∼94%) and storage temperature is −25 to −15°C,^136^, ^138^, ^140^, ^141^ Johnson and Johnson's vaccine is based on a replication‐incompetent adenovirus 26 vector that encodes a stabilized S protein.^136^ This vaccine requires refrigeration (2 to 8°C).^138^ Only one dose of this vaccine is 66% effective in preventing moderate to severe COVID‐19 and 100% effective in preventing COVID‐19‐related hospitalization and death.^142^ AstraZeneca/Oxford vaccine, which requires refrigeration (2 to 8°C), is based on a replication‐incompetent chimpanzee adenovirus vector that expresses the S protein.^136^, ^138^ It has 76% efficacy against symptomatic COVID‐19, while 100% efficacy against severe or critical disease in addition to hospitalizations.^143^ Sinovac is an inactivated vaccine with an aluminum hydroxide adjuvant.^136^ The storage temperature is 2 to 8°C.^138^ This vaccine showed 50.7% efficacy in Brazil,^144^ and 83.5% efficacy in Turkey.^145^ Sinopharm is inactivated, whole‐virus vaccine^136^ with 79% efficacy against symptomatic SARS‐CoV‐2 infection 14 or more days after the second dose.^143^ Covaxin (BBV152), a whole‐virion inactivated Vero cell‐derived platform, has an aluminum hydroxide and a TLR agonist adjuvant. This vaccine, which is stable at 2–8°C, results in an efficacy of 80.6%.^136^, ^146^, ^147^ Covovax is a recombinant S protein nanoparticle vaccine with 89.3% efficacy.^147^ Nuvaxovid (NVX‐CoV2373), another recombinant S protein nanoparticle‐based vaccine, includes the full‐length S protein and a saponin‐based Matrix‐M adjuvant.^148^ The efficacy of Nuvaxovid to prevent the onset of COVID‐19 from 7 days after the second dose is reported as 90.4%.^149^ Besides these, as of December 2, 2022, 50 vaccines have been approved by at least one country (Table 4).^106^

**TABLE 4:** COVID‐19 vaccines approved by at least one country.^106^

**Vaccine name**	**Vaccine platform**	**Manufacturer**	**Number of countries approving**	**Number of trials**
Zifivax (ZF2001)	Protein subunit	Anhui Zhifei Longcom, Beijing, People's Republic of China	4 countries	21 trials in 5 countries
Noora vaccine	Protein subunit	Bagheiat‐Allah University of Medical Sciences, Tehran, Iran	1 country	3 trials in 1 country
Covaxin (BBV152)	Inactivated	Bharat Biotech, Hyderabad, India	14 countries	16 trials in 2 countries
iNVOVACC	Nonreplicating viral vector	Bharat Biotech, Hyderabad, India	1 country	4 trials in 1 country
Corbevax (BECOV2A)	Protein subunit	Biological E Limited, Hyderabad, India	2 countries	7 trials in 1 country
Convidecia Air (Ad5‐nCoV‐IH)	Nonreplicating viral vector	CanSino, Tianjin, People's Republic of China	2 countries	5 trials in 4 countries
Convidecia (Ad5‐nCoV)	Nonreplicating viral vector	CanSino, Tianjin, People's Republic of China	10 countries	14 trials in 6 countries
Abdala (CIGB‐66)	Protein subunit	Cuban Center for Genetic Engineering and Biotechnology, Havana, Republic of Cuba	6 countries	5 trials in 1 country
KoviVac	Inactivated	Chumakov Federal Scientific Center for Research and Development of Immune‐and‐Biological Products of Russian Academy of Sciences, Moscow, Russian Federation	3 countries	3 trials in 1 country
Gam‐COVID‐Vac (Sputnik, rAd5)	Nonreplicating viral vector	Gamaleya National Center of Epidemiology and Microbiology, Moscow, Russian Federation	1 country	2 trials in 0 countries
Sputnik Light	Nonreplicating viral vector	Gamaleya National Center of Epidemiology and Microbiology, Moscow, Russian Federation	26 countries	7 trials in 3 countries
Sputnik V (Gam‐COVID‐Vac)	Nonreplicating viral vector	Gamaleya National Center of Epidemiology and Microbiology, Moscow, Russian Federation	74 countries	25 trials in 8 countries
GEMCOVAC‐19	RNA	Gennova Biopharmaceuticals Limited, Maharashtra, India	1 country	2 trials in 1 country
Turkovac (ERUCOV‐VAC)	Inactivated	Health Institutes of Turkey, Istanbul, Turkey	1 country	8 trials in 1 country
Soberana 02 (FINLAY‐FR‐2, Pastu Covac)	Protein subunit	Instituto Finlay de Vacunas Cuba, Havana, Republic of Cuba	4 countries	7 trials in 2 countries
Soberana Plus (FINLAY‐FR‐1A)	Protein subunit	Instituto Finlay de Vacunas Cuba, Havana, Republic of Cuba	2 countries	5 trials in 1 country
Jcovden (Ad26.COV2.S)	Nonreplicating viral vector	Janssen (Johnson & Johnson), Beerse, Belgium	113 countries	26 trials in 25 countries
V‐01	Protein subunit	Livzon Mabpharm Inc., Guanghong, China	1 country	7 trials in 3 countries
Covifenz (CoVLP, MT‐2766, Plant‐based virus‐like particle [VLP])	VLP	Medicago, Quebec City, Canada	1 country	6 trials in 6 countries
MVC‐COV1901	Protein subunit	Medigen, Taipei, Taiwan, People's Republic of China	4 countries	15 trials in 4 countries
Spikevax (mRNA‐1273, Elasomeran)	RNA	Moderna, Cambridge, MA, USA	88 countries	70 trials in 24 countries
Spikevax Bivalent Original/Omicron BA.1	RNA	Moderna, Cambridge, MA, USA	38 countries	5 trials in 4 countries
Spikevax Bivalent Original/Omicron BA.4/BA.5	RNA	Moderna, Cambridge, MA, USA	33 countries	2 trials in 1 country
Recombinant SARS‐CoV‐2 Vaccine (CHO Cell, NVSI‐06‐08)	Protein subunit	National Vaccine and Serum Institute, Beijing, People's Republic of China	1 country	3 trials in 2 countries
Nuvaxovid (NVX‐CoV2373)	Protein subunit	Novavax, Gaithersburg, MD, USA	40 countries	22 trials in 14 countries
FAKHRAVAC (MIVAC)	Inactivated	Organization of Defensive Innovation and Research, Tehran, Iran	1 country	3 trials in 1 country
Vaxzevria (AZD1222, ChAdOx1 nCoV‐19)	Nonreplicating viral vector	Oxford University/AstraZeneca, Södertälje, Sweden	149 countries	73 trials in 34 countries
Comirnaty (BNT162b2, Tozinameran)	RNA	Pfizer/Biontech, Mainz, Germany	149 countries	100 trials in 31 countries
Comirnaty Bivalent Original/Omicron BA.1	RNA	Pfizer/Biontech, Mainz, Germany	35 countries	3 trials in 5 countries
Comirnaty Bivalent Original/Omicron BA.4/BA.5	RNA	Pfizer/Biontech, Mainz, Germany	33 countries	4 trials in 1 country
IndoVac	Protein subunit	PT Bio Farma, Jawa Barat Indonesia	1 country	4 trials in 1 country
Razi Cov Pars	Protein subunit	Razi Vaccine and Serum Research Institute, Karaj, Iran	1 country	5 trials in 1 country
QazVac (QazCovid‐in)	Inactivated	Research Institute for Biological Safety Problems, Guardeyskiy, Republic of Kazakhstan	2 countries	3 trials in 1 country
VidPrevtyn Beta	Protein subunit	Sanofi/GSK	30 countries	3 trials in 2 countries
Covishield (Oxford/ AstraZeneca formulation)	Nonreplicating viral vector	Serum Institute of India, Pune, India (based on AstraZeneca technology)	49 countries	6 trials in 1 country
COVOVAX (Novavax formulation)	Protein subunit	Serum Institute of India, Pune, India (based on Novavax technology)	6 countries	7 trials in 3 countries
KCONVAC (SARS‐CoV‐2 Vaccine (Vero Cells), KconecaVac	Inactivated	Shenzhen Kangtai Biological Products Co., Shenzhen, China	2 countries	7 trials in 1 country
COVIran Barekat (COVID‐19 Inactivated Vaccine)	Inactivated	Shifa Pharmed Industrial Co, Karaj, Iran	1 country	6 trials in 1 country
Covilo (BBIBP‐CorV (Vero Cells))	Inactivated	Sinopharm (Beijing), People's Republic of China	93 countries	39 trials in 18 countries
Inactivated (Vero Cells)	Inactivated	Sinopharm (Wuhan), People's Republic of China	2 countries	9 trials in 7 countries
CoronaVac	Inactivated	Sinovac, Beijing, People's Republic of China	56 countries	42 trials in 10 countries
SKYCovione	Protein subunit	SK Bioscience Co. Ltd., Republic of Korea	1 country	7 trials in 6 countries
TAK‐019 (NovaVax formulation)	Protein subunit	Takeda, Tokyo, Japan (based on Moderna technology)	1 country	3 trials in 1 country
TAK‐919 (Moderna formulation)	RNA	Takeda, Tokyo, Japan (based on Moderna technology)	1 country	2 trials in 1 country
VLA2001	Inactivated	Valneva, Saint‐Herblain, France	33 countries	9 trials in 4 countries
SpikoGen (COVAX‐19)	Protein subunit	Vaxine/CinnaGen Co., Tehran, Iran	1 country	8 trials in 2 countries
Aurora‐CoV (EpiVacCorona‐N)	Protein subunit	“Vector,” National Research Center for Virology and Biotechnology, Novosibirsk, Russian Federation	1 country	2 trials in 1 country
EpiVacCorona	Protein subunit	“Vector,” National Research Center for Virology and Biotechnology, Novosibirsk, Russian Federation	4 countries	4 trials in 1 country
AWcorna	RNA	Walvax, Yunnan, China	1 country	4 trials in 3 countries
ZyCoV‐D	DNA	Zydus Cadila, Ahmedabad, India	1 country	6 trials in 1 country

#### 7.3.1 Efficacy and effectiveness of vaccines against SARS‐CoV‐2 variants

According to a trial of Novavax (NVX‐CoV2373) vaccine, a post hoc analysis demonstrated that it has an efficacy of 86.3% against Alpha variant (Lineage B.1.1.7).^150^ When Alpha variant (Lineage B.1.1.7) was dominant in Israel, Pfizer‐BioNTech (BNT162b2) vaccine demonstrated 92% protection against asymptomatic infection, 97% against severe disease, and 97% against symptomatic. Pfizer‐BioNTech (BNT162b2) vaccine and AstraZeneca vaccine showed 85% and 94% reduction in hospitalizations, respectively, against Alpha variant (Lineage B.1.1.7) in Scotland.^151^ The effectiveness of the Pfizer‐BioNTech (BNT162b2) vaccine against the severe disease was 97.4%, while against any documented infection with the Alpha variant (Lineage B.1.1.7) was 89.5% in Qatar.^152^ Vaxzevria (AZD1222) was 70% effective against Alpha variant (Lineage B.1.1.7) infection as determined by nucleic acid amplification tests, while the effectiveness of the vaccine was 77% against lineages other than Alpha variant (Lineage B.1.1.7).^153^

The protection after the first dose of the Pfizer‐BioNTech (BNT162b2) vaccine was decreased in Qatar. In addition, after the second dose, it showed 97.4% protection against severe disease and 75% effectiveness against any documented infection with Beta variant (Lineage B.1.351).^152^ J&J/Janssen's vaccine (Ad26.COV2.S) clinical trial demonstrated 65%–66% protection against hospitalization, while 91%–95% protection against mortality in South Africa. These efficacy results are similar to studies conducted in the United States.^154^ Also, another clinical trial showed that Novavax provided 60% protection against the Beta variant (Lineage B.1.351).^155^ Additionally, a significant decrease in the neutralization of Beta variant (Lineage B.1.351) by sera from humans vaccinated with Moderna (mRNA‐1273) was detected; however, there was no significant impact against Alpha variant (Lineage B.1.1.7).^156^

In Brazil, where the Gamma variant (Lineage P1) was predominant, comparable efficacy to the J&J/Janssen vaccination was reported. Also, comparable real‐world effectiveness was demonstrated by the Sinovac vaccination (CoronaVac) and the AstraZeneca vaccination against symptomatic COVID‐19 during wide Gamma variant (Lineage P1) circulation.^154^

In England, vaccine effectiveness of 67% and 88% against symptomatic disease following infection with Delta variant (Lineage B.1.617.2) has been noted after two doses of Vaxzevria (AZD1222) and Pfizer‐BioNTech (BNT162b2), respectively.^157^ In addition, in Scotland, Vaxzevria (AZD1222) and Pfizer‐BioNTech (BNT162b2) vaccines were effective to decrease the risk of infection and also hospitalization against Delta variant (Lineage B.1.617.2). But the level of protection was not as high as against Alpha variant (Lineage B.1.1.7).^158^ In Qatar, the effectiveness against the Delta variant (Lineage B.1.617.2) was 86% with Moderna (mRNA‐1273) and 60% with BioNTech (BNT162b2) vaccines.^151^

Two shots of the Pfizer‐BioNTech (BNT162b2) vaccines have been reported to be 70% effective in preventing hospitalization from infection with the Omicron variant (Lineage B.1.1.529).^159^ On the other hand, a third dose of the Pfizer‐BioNTech (BNT162b2) vaccine has been shown to neutralize the Omicron variant (Lineage B.1.1.529). Different studies have shown that CoronaVac and Moderna (mRNA‐1273) and Pfizer/BioNTech (BNT162b2) mRNA vaccines have significantly decreased neutralizing activity against the Omicron variant (Lineage B.1.1.529).^160^

## 8 DRUG DEVELOPMENT STUDIES FOR COVID‐19

Anticoronavirus therapy is divided into two categories according to the target. One of this therapy acts on the coronavirus itself, and the other is the human immune system. The human immune system plays a significant role in controlling the infection and replication of coronavirus. Therefore, blocking the human signaling pathways required for virus replication might show a particular antiviral effect.^161^ The therapies that act on the coronavirus itself contain blocking the binding of the virus to the human cell receptors, preventing the synthesis of viruses’ RNA, inhibiting processing of the viral assembly through acting on some structural proteins, or inhibiting viral replication via acting on crucial enzymes of the virus.^162^

Researchers have enhanced three strategies for developing the drug against coronavirus.^27^ One of them is to test existing broad‐spectrum antiviral agents. In this strategy, interferons, cyclophilin inhibitors, and ribavirin are used to treat coronavirus pneumonia. Their advantages are that their potential efficacy, metabolic characteristics, side effects, and applied dose are clearly known because they have been approved for treating viral infections. However, they cannot kill coronavirus in a targeted manner.^162^ In the second strategy, the existing molecular databases are used for high‐throughput screening of specific molecules, which may have a therapeutic effect on coronavirus. Thus, new functions of drug molecules could be identified. In the third strategy, pathological characteristics and genomic information of different CoVs are determined to develop new targeted drugs, which would show better therapeutic effects.^163^ It should not be forgotten that the research procedure for new drugs may take several years.^162^

As of January 21, 2023, 8842 COVID‐19 studies, completed or still in progress, are listed on ClinicalTrials.gov.^164^ Different target drugs have been identified to date, and various immunomodulators and antiviral molecules were applied to patients with severe COVID‐19. These antiviral molecules include nucleoside analogs such as remdesivir and molnupiravir, protease inhibitors such as lopinavir/ritonavir, antibiotics such as azithromycin, antimalarial such as hydroxychloroquine, nAbs such as sotrovimab, antiparasitic such as ivermectin. On the other hand, immunomodulators contain antibodies such as convalescent plasma and tocilizumab anti‐IL‐6 (Interleukin‐6) receptor mAb, interferon such as IFNb‐1a (Interferon beta‐1a) and peg‐IFNλ‐1 (Interferon lambda‐1), corticosteroids such as dexamethasone and inhaled budesonide, anticoagulants such as heparin, angiotensin receptor blocker such as telmisartan.^165^

In addition to viral polymerase and the M^pro^, the PL^pro^ was considered to have significant potential as therapeutic targets.^166^ The SARS‐CoV‐2 PL^pro^ mediates the cleavage of viral polyprotein and also modulates the innate immune response of the host upon viral infection. PL^pro^ has deISGylating and deubiquitinating activities, which are implicated in suppressing innate immune responses and promoting viral replication. It can remove *ISG15* (interferon‐stimulated gene 15) and ubiquitin modifications from host proteins.^167^ The active site of PL^pro^ is now the subject of the most research because of its significance, yet due to its structural characteristics, the active site itself has proven to be quite a difficult target for rational drug design.^166^

**FIGURE 4:**
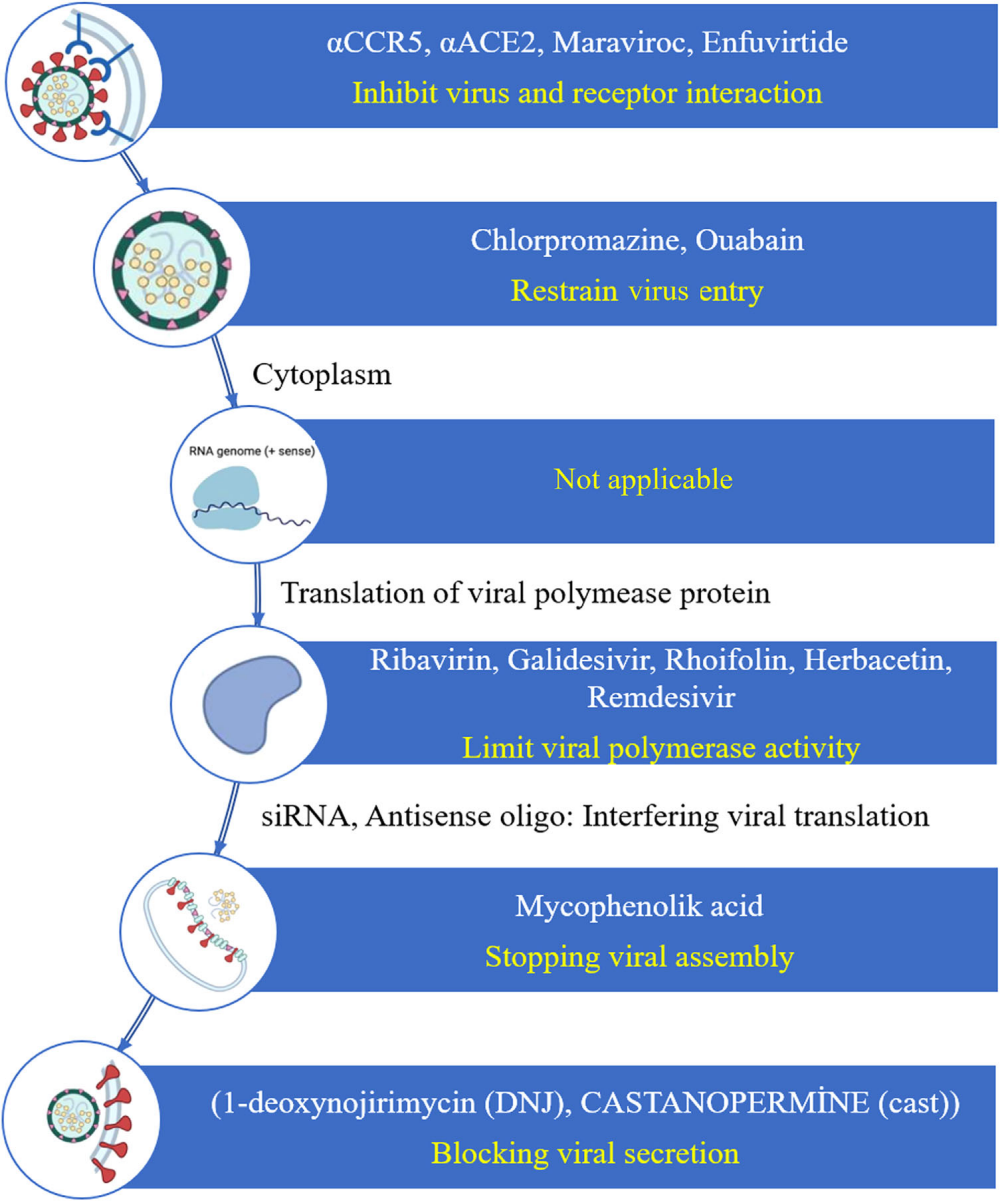
Virus replication cycle and the steps that are targeted by antiviral drugs. PCR, polymerase chain reaction; RNA, ribonucleic acid.

Antiviral drugs with the capacity to prevent virus attachment and entry by specifically targeting the S protein and cell surface protease could block the virus replication cycle and may be used as treatment approaches for COVID‐19 patients^168^, ^169^ (Figure 4). The FDA has now issued an emergency use authorization for three antiviral drugs.^170^ Remdesivir, which can block the activity of RNA‐dependent RNA polymerase with in vitro inhibitory activity against SARS‐CoV, MERS‐CoV, and SARS‐CoV‐2, was reported early as a promising therapeutic candidate for COVID‐19 infection.^171^ Therefore, in May 2020, the FDA approved remdesivir as the first drug to treat COVID‐19 in adults and children requiring hospitalization.^172^ However, the clinical application of remdesivir has been highly restricted because of the requirement of intravenous administration, variable antiviral activity in different organelles, and unstable concentrations in plasma. Moreover, it did not reduce the mortality caused by COVID‐19.^173^, ^174^ On the other hand, molnupiravir, which is an oral antiviral drug with β‐D‐*N4‐*hydroxycytidine as the active ingredient, has been used since December 2021. Molnupiravir shows antiviral activity by causing catastrophic mutations during viral RNA replication using RNA‐dependent RNA polymerase.^175^, ^176^ The other antiviral drug is nirmatrelvir/ritonavir (Paxlovid), which inhibits the M^pro^, 3CL protease, of SARS‐CoV‐2 via reversible covalent bonding between the catalytic cysteine (Cys145) of M^pro^ and the nitrile warhead of nirmatrelvir. This drug has been used since December 2021 and has extensive antiviral activity and good off‐target selectivity.^176^, ^177^, ^178^

In order to accelerate the development of antiviral drugs to combat the pandemic, it is essential to predict drug resistance before it becomes prevalent in clinical settings.^179^ Drug resistance against remdesivir and nirmatrelvir has been reported in some studies. RdRp mutations that confer resistance to remdesivir are well known through in vitro studies. Numerous SARS‐CoV‐2 sequencing efforts have allowed to assess emergence and fitness of antiviral resistance in vivo.^180^ The *de novo* emergence of remdesivir resistance mutation, E802D, has been reported in an immunocompromised patient with acquired B‐cell deficiency, who had persistent SARS‐CoV‐2 infection. In vitro tests revealed that this mutation conferred an approximately six‐fold increase in remdesivir IC_50_; however, resulted in a fitness cost in the absence of remdesivir.^181^ In another study, 100 naturally occurring M^pro^ mutations, which are located at the nirmatrelvir binding site, have been studied. Twenty of these mutants demonstrated comparable enzymatic activity to the wild type (10‐fold change) and resistance to nirmatrelvir (> 10‐fold increase).^179^

Since viruses need host factors to translate their transcripts, targeting the host factor(s) are important for developing novel antiviral drugs.^182^ Host‐targeting antivirals (HTAs) consist reagents which target the host proteins that are involved in the life cycle of virus, regulating the function of the immune system or other cellular processes in host cells.^183^ Additionally, a genetic barrier to resistance is produced via the minimal diversity of host components that HTAs target.^182^ Even before comprehensive information about a virus becomes available, host‐targeting drugs can be used to combat newly developing infections.^184^

Also, cases of Paxlovid rebound have been reported where mild COVID‐19 symptoms recur after a period of initial recovery post‐treatment with Paxlovid. According to a preliminary study, this occurrence is likely caused by insufficient drug exposure because infected cells that are not exposed to the drug would allow the virus to continue replicating and symptoms to reappear.^185^, ^186^, ^187^ These rebound cases are still rare, though, as in the Paxlovid clinical study, < 1% of 5287 patients required hospitalization or care in the emergency department for COVID‐19 symptoms 5–15 days following Paxlovid treatment.^185^, ^188^

To date, convalescent plasma, classic adaptive immunotherapy, has been exerted to prevent and treat various infectious diseases. The FDA has approved using blood plasma from patients that have recovered from COVID‐19 infection with a high nAb titer because of being an essential source of convalescent plasma.^189^ Besides the optimal dose, the treatment time point is also significant. Studies have shown that convalescent plasma therapy has low risk and a potential therapeutic effect for severe COVID‐19 patients.^190^ However, there was a lack of procedure control and standardization regarding the level of antibodies in convalescent plasma units and the donor selection process. These issues may lead to various therapeutic effects in patients with the same disease.^191^

Antibody therapies for COVID‐19 also contain hyperimmune gamma globulin as well as mAbs preparations.^192^ Production of hyperimmune globulin has been pursued in parallel with research into convalescent plasma during COVID‐19. Hyperimmune globulin is a licensed product made from convalescent plasma from thousands of donors. Hyperimmune globulin includes concentrated polyclonal immune globulin fraction and is subject to pathogen reduction techniques.^193^

On the other hand, several mAb, which recognize a single antigen, have been discovered to bind the SARS‐CoV‐2 S protein and block this virus from invading human cells. An mAb may be administered intravenously to COVID‐19 patients, usually at an emergency department, an infusion center, or another outpatient settings—such as a nursing home or patient's home. Some potential risks of mAbs including allergic or nonallergic infusion‐related reaction have been reported. mAbs are intended for patients just been diagnosed with COVID‐19 who have some risk factors for severe infection but who are not sick enough to be hospitalized.^194^ Thus, results from current trials do not show a benefit of mAbs in the majority of hospitalized patients.^195^

Antibody‐based COVID‐19 therapies have demonstrated promising clinical efficacy and safety. To date, different nAbs have been approved by the FDA for urgent treatment of mild to moderate COVID‐19. Some nAbs such as imdevimab and casirivimab target viral RBD and have demonstrated strong neutralizing activity against wild‐type SARS‐CoV‐2 infection. On the other hand, Omicron variants exhibit notable resistance to the majority of approved nAbs such as bamlanivimab, casirivimab, imdevimab, and regdanvimab.^196^

## 9 CONCLUSION AND PERSPECTIVES

In the 21st century, due to human activities and rapid globalization, the risk of viral transmission has increased across continents, and various pandemics have been observed. The latest pandemic, COVID‐19, has affected nearly every aspect of our lives. We have been forced to adapt to the new normal such as quarantine, working from home, having to homeschool, wearing face masks, and so forth. In addition, the global prevalence of depression and anxiety has increased. 3 years have passed since this pandemic, which claimed more than six million lives and affected billions. COVID‐19 continues to threaten us for reasons such as new variants of SARS‐CoV‐2, high transmissibility, some people not being vaccinated, and not using personal protective equipment. Early and accurate diagnoses are essential to control infectious diseases. Various diagnoses, vaccines, and drug studies have been carried out to support the fight against the COVID‐19 pandemic. The presence of SARS‐CoV‐2 has been determined by molecular techniques such as RT‐PCR, RT‐LAMP, CRISPR, amplicon‐based metagenomic sequencing, and nucleic acid hybridization using microarray and serological techniques such as lateral flow test and ELISA. The fact that this disease shows symptoms similar to other viral infections leads people to think they do not carry the SARS‐CoV‐2 virus, even if they have it. In addition, those with the suspected disease do not prefer to go for testing because they are afraid of the risk of contamination in the hospital. Therefore, people have begun to use COVID‐19 self‐test kits, which give rapid results and are easy to use. However, these serological tests have low accuracy and may give false positive or false negative results. In such cases, the need to develop nucleic acid‐based rapid diagnostic kits with high specificity and sensitivity, which are not on the market, has emerged.

On the other hand, numerous scientists and pharmaceutical companies have tried to develop safe and effective vaccines against COVID‐19 infection, and 50 vaccines have been approved by at least one country. The primary concern in vaccine studies is the continuous mutation in SARS‐CoV‐2. The Omicron variant, detected recently, is less virulent but more infectious than previous SARS‐CoV‐2 variants. Unfortunately, COVID‐19 continues to run its course. Omicron will most likely not be the last COVID‐19 variant. In the future, the currently available vaccines may be less effective in fighting new emerging variants. Although several clinical trials have been carried out until now, it is still urgent to develop highly effective and low‐toxic antiviral drugs that can control the SARS‐CoV‐2 infection. Moreover, developing rapid diagnostic kits for different variants including the Omicron sublineages with which patients are infected is important to guide the selection of antibody drugs. To assess the therapeutic potential of combining PL^pro^ inhibitors with RdRp or M^pro^ inhibitors, combination experiments may be planned. On the other hand, due to COVID‐19 and the threat of bacterial coinfections, antibiotic use has increased, perhaps contributing to the development of drug resistance. This pandemic has taught that new strategies must be developed to combat a possible pandemic, such as (i) quickly obtaining and sharing the genetic sequences of viruses as they emerge, (ii) production of vaccines, therapeutics, and diagnostic kits in regional center that will enable them to be easily distributed everywhere, and (iii) building plug‐and‐play technical platforms such as rapidly modifying mRNA technology or adenovirus vectors.

## AUTHOR CONTRIBUTIONS

İ.P. performed writing and editing original draft, conceptualization and investigation. T.O.O. performed writing and editing original draft, conceptualization, and investigation. I.D. performed writing original draft. S.O. performed writing original draft. All authors have read and approved the final manuscript.

## CONFLICT OF INTEREST STATEMENT

The authors declare no conflicts of interest.

## FUNDING INFORMATION

No financial support was received from any institution or organization in the writing of this review article.

## ETHICS STATEMENT

This review does not require an ethical statement.

## Supporting information

Supporting Information

## Data Availability

Not applicable.
