# Supplementary material for: COVID‐19 in early 2023: Structure, replication mechanism, variants of SARS‐CoV‐2, diagnostic tests, and vaccine & drug development studies
Source: MedComm (2020). 2023 Apr 8;4(2):e228. doi: 10.1002/mco2.228 (PMC10082934; doi:10.1002/mco2.228)
Supplement: Supplementary file 1 — Supporting Information [file MCO2-4-e228-s001.docx]

**COVID-19 in early 2023:** **Structure, Replication Mechanism, Variants of SARS-CoV-2, Diagnostic Tests, Vaccine and Drug Development Studies**

**Running Title: COVID-19 in early 2023**

İlker Polatoğlu^1*^, Tulay Oncu-Oner^1^, Irem Dalman^2^, Senanur Ozdogan^1^

^1^Department of Bioengineering, Manisa Celal Bayar University, Yunusemre, 45140 Manisa, Turkey

^2^Department of Bioengineering, Ege University, Bornova, 35040 Izmir, Turkey

*Corresponding Author: Dr. Ilker Polatoğlu

Address: Department of Bioengineering, Manisa Celal Bayar University, Yunusemre, 45140 Manisa, Turkey

Tel: +90 236 201 24 53

e-mail: [ilker.polatoglu@cbu.edu.tr](mailto:ilker.polatoglu@cbu.edu.tr)

e-mail: İlker Polatoğlu: [ilker.polatoglu@cbu.edu.tr](mailto:ilker.polatoglu@cbu.edu.tr), Tulay Oncu-Oner: [tulay.oncu@cbu.edu.tr](mailto:tulay.oncu@cbu.edu.tr), Irem Dalman: [iremmdalman@gmail.com](mailto:iremmdalman@gmail.com), Senanur Ozdogan: sn.nr9660@gmail.com

ORCID ID: İlker Polatoğlu: 0000-0002-6099-062X; Tulay Oncu-Oner: 0000-0001-6180-5409

**Supplementary Files**


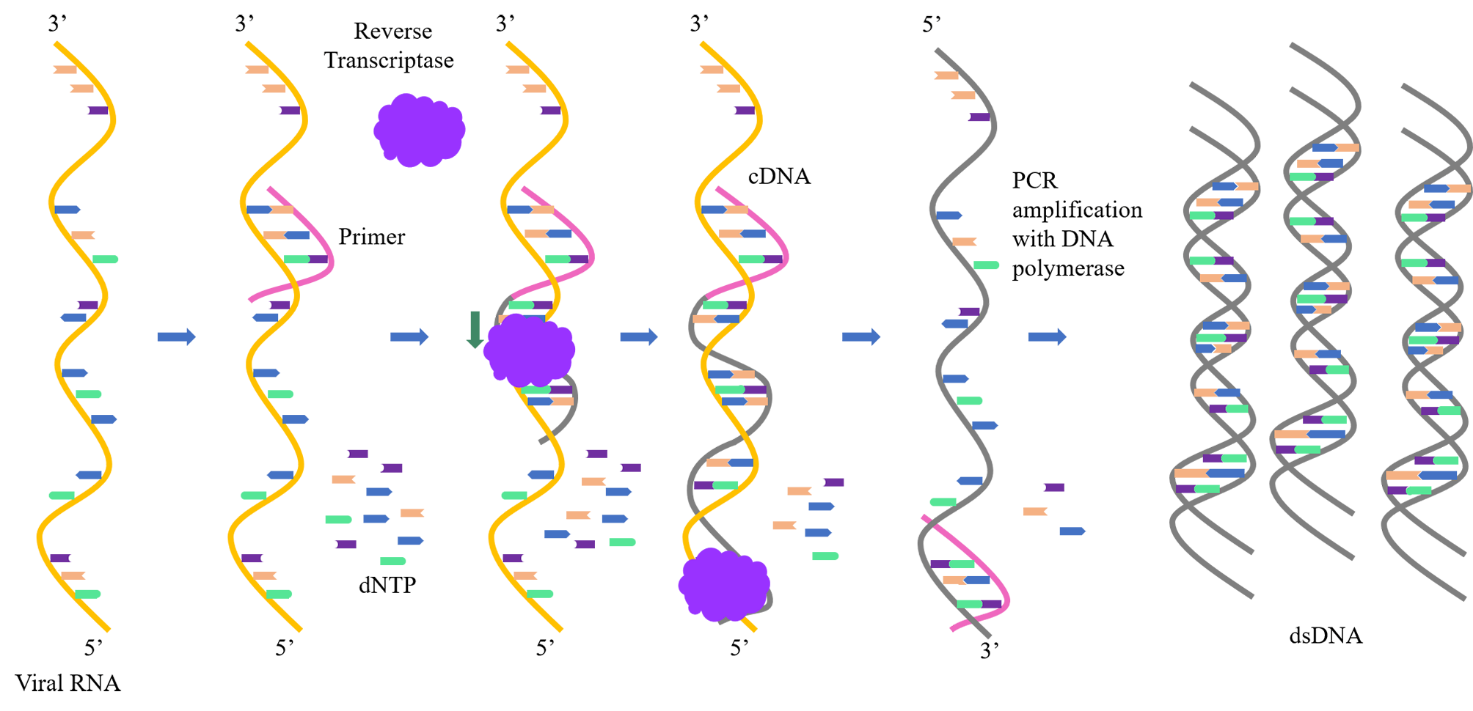


**FIGURE S1.** The working principle of RT-PCR for viral RNA detection.


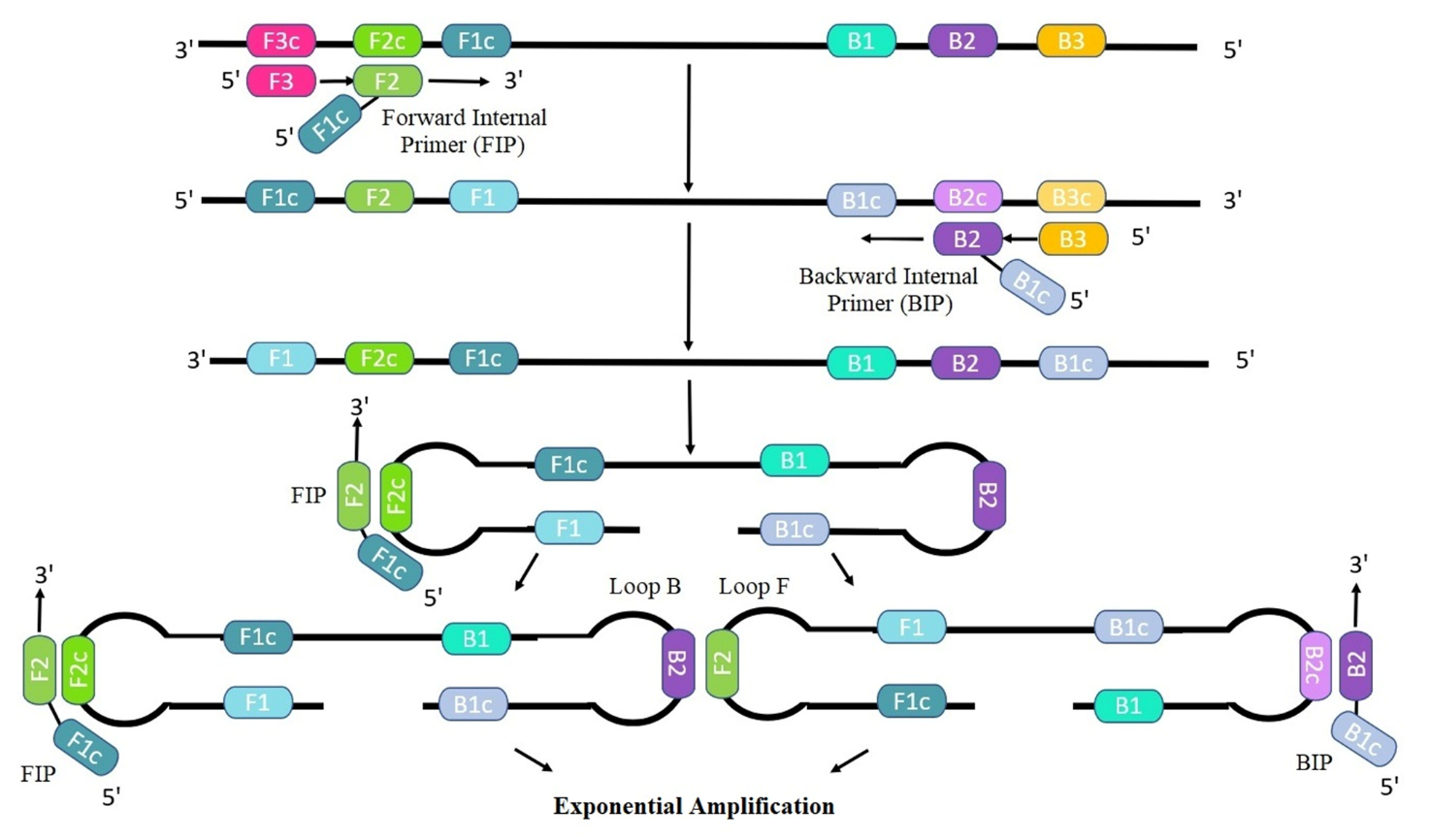


**FIGURE S2.** Schematic representation of the loop-mediated isothermal amplification. Reproduced with permission from Ref 40, 2015, Copyright 2015 Elsevier.


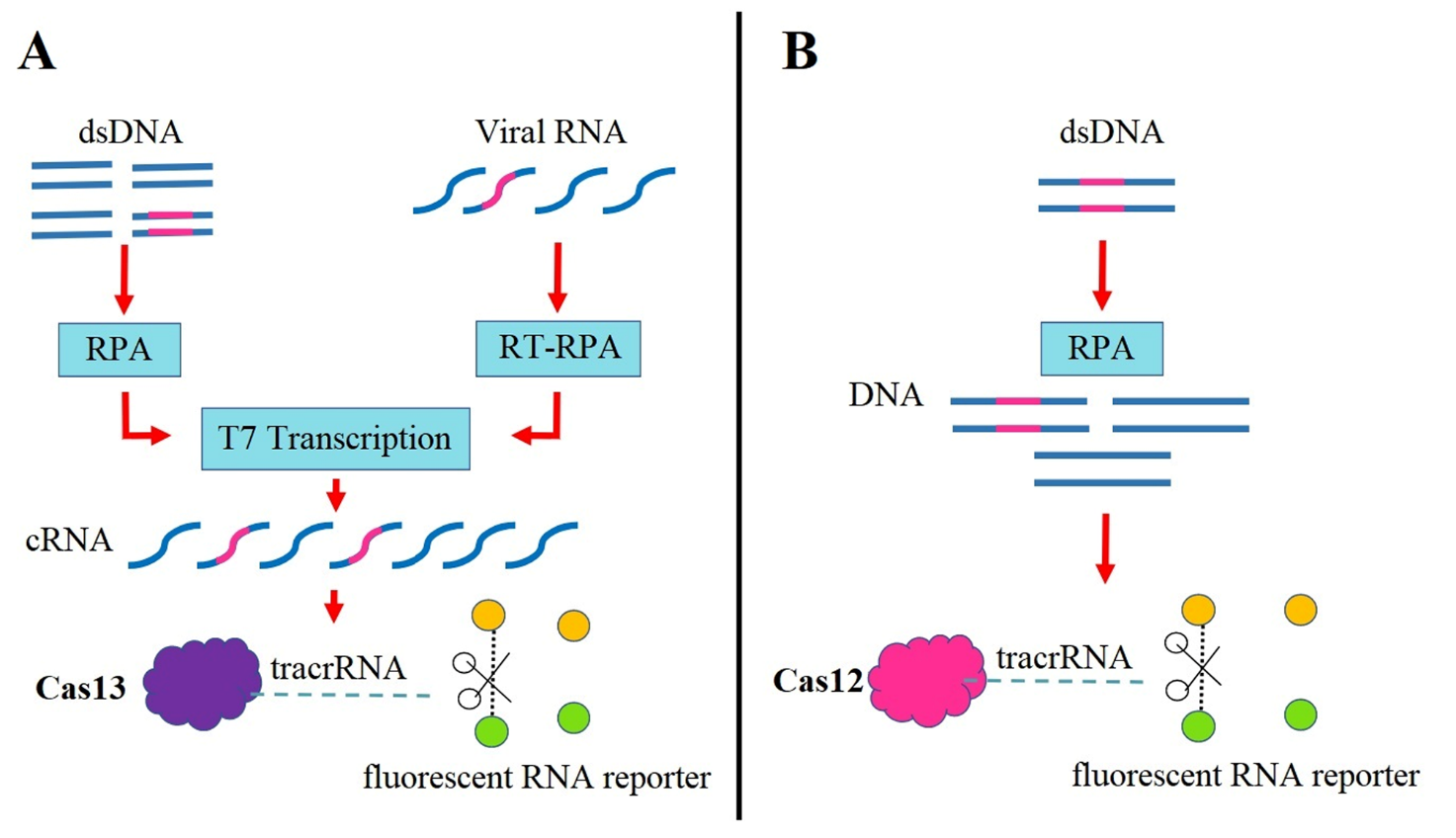


**FIGURE S3.** Schematic representation of (A) SHERLOCK technology and (B) DETECTR technology.


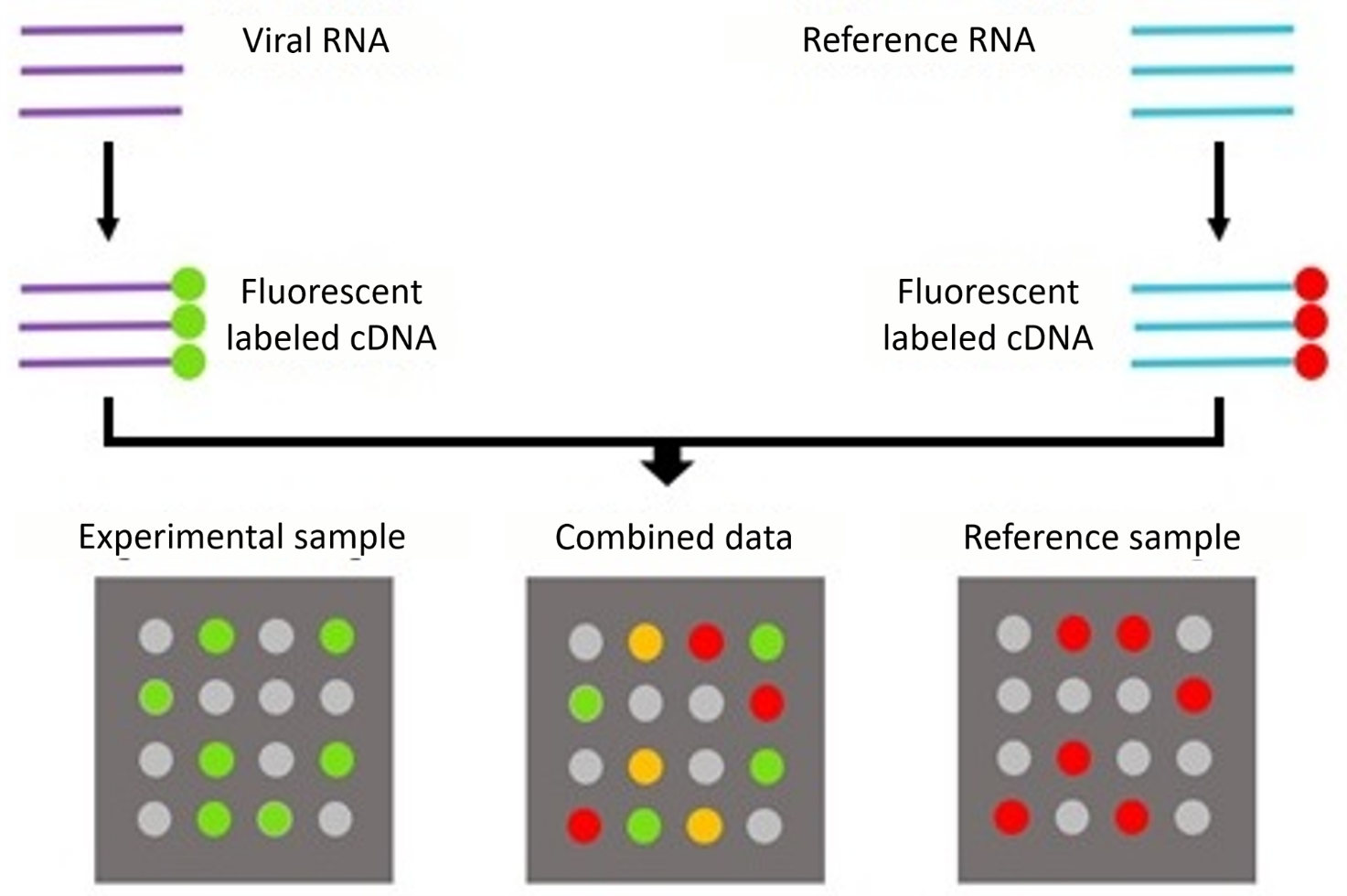


**FIGURE S4.** Schematic representation of nucleic acid hybridization using microarray platform. Reproduced with permission from Ref 29, 2020, Copyright 2020 American Chemical Society


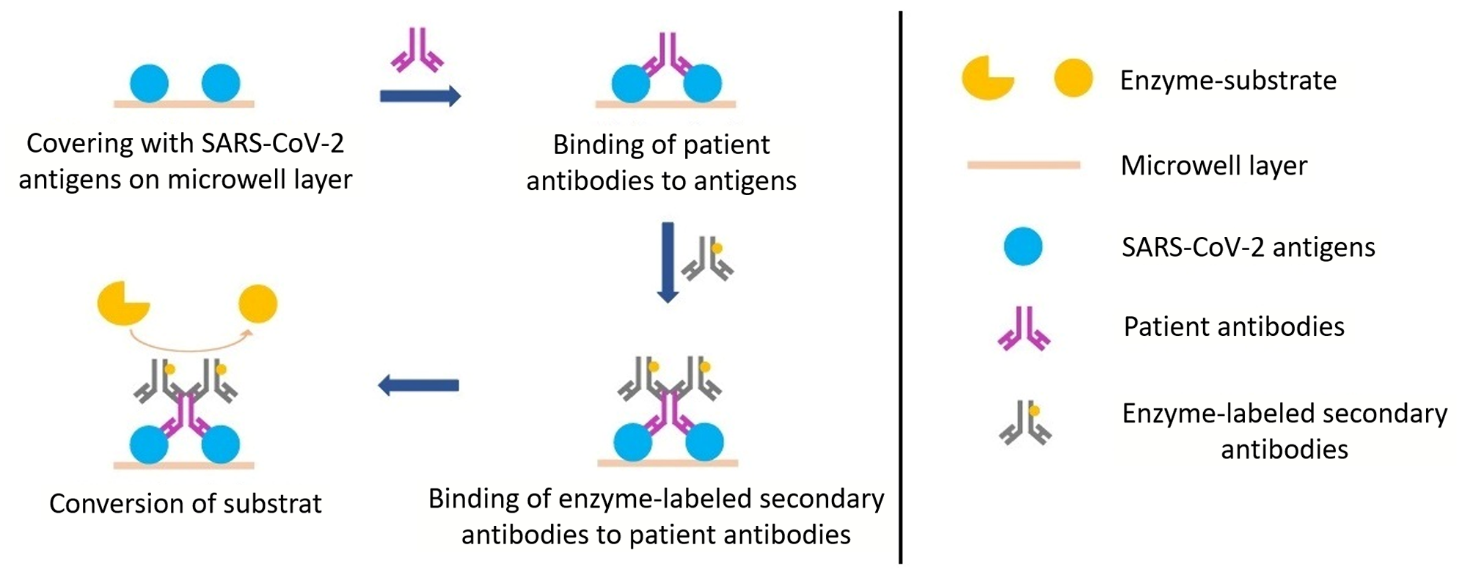


**FIGURE S5.** Schematic representation of indirect ELISA used to detect anti-SARS-CoV-2 antibodies. Reproduced with permission from Ref 29, 2020, Copyright 2020 American Chemical Society


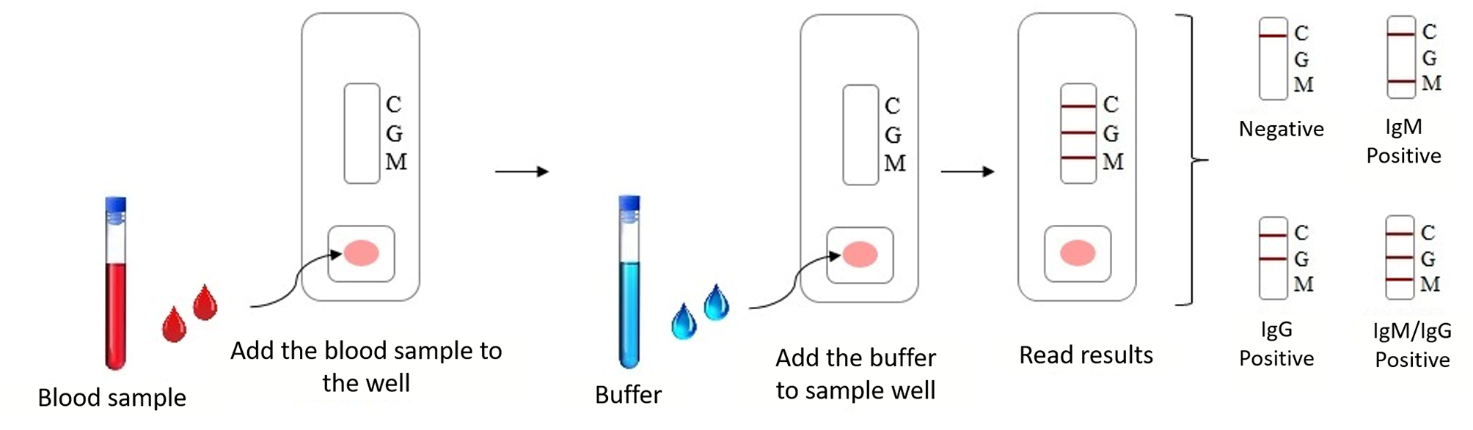


**FIGURE S6.** The working principle of rapid IgM/IgG combined antibody test for COVID-19.
